# Identification and characterization of quorum-quenching enzyme AiiF from *Acidithiobacillus ferrooxidans* and its application for biofilm disruption

**DOI:** 10.1128/aem.00758-26

**Published:** 2026-07-17

**Authors:** Mallori A. Herishko, Hannah S. Zurier, Scott Banta

**Affiliations:** 1 Department of Chemical Engineering, Columbia University 5798, New York, New York, USA; Indiana University Bloomington, Bloomington, Indiana, USA

**Keywords:** quorum sensing, quorum quenching, lactonase, biofilm, bioleaching, *Acidithiobacillus ferrooxidans*, *Acidithiobacillus thiooxidans*

## Abstract

Quorum sensing-induced functions, including biofilm formation, can greatly influence biotechnology applications, including industrial metal bioleaching processes. Biofilm formation by the acidophilic chemolithoautotrophs *Acidithiobacillus ferrooxidans* and *Acidithiobacillus thiooxidans* is a key factor affecting mineral attachment and leaching efficiency, and is mediated by *N*-acyl homoserine lactone (AHL) quorum-sensing systems. Here, we identified an AHL lactonase gene native to *A. ferrooxidans* strains TFBk and BY-3, termed AiiF, which suppresses AHL-dependent quorum sensing. The kinetic parameters of AHL degradation by AiiF recombinantly expressed in *Escherichia coli* were obtained and represent a relatively slow reaction (*K*_m_ of 3.5 ± 2.2 mM and *k*_cat_ of 0.15 ± 0.04 $s^{-1}$), where the high *K*_m_ value is similar to its closest relative, AiiA. AHL docking simulations and *in vivo* biosensor data suggest that AiiF is mechanistically promiscuous and exhibits broad substrate specificity. Exogenous addition of AiiF disrupted biofilm formation in laboratory strains of both *A. ferrooxidans* and *A. thiooxidans*. Overexpression of recombinant AiiF in *A. thiooxidans* demonstrated that quorum quenching can reduce biofilm formation and influence growth behavior on a sulfur substrate.

**IMPORTANCE:**

Biomining of critical materials with metal sulfide-oxidizing bacteria can reduce costs and environmental impacts. The relationship between biofilm formation and bioleaching efficiency is complex, with different mineral systems being enhanced or inhibited by biofilm formation. We identified and characterized a new lactonase enzyme native to *Acidithiobacillus ferrooxidans*, AiiF, that can degrade *N-*acyl homoserine lactone (AHL) autoinducers. This enzyme can reduce biofilm formation in bioleaching microbes, and this activity may be leveraged to enhance future biomining activities.

## INTRODUCTION

Quorum sensing (QS) is a cell-density-dependent regulatory mechanism that enables bacterial communities to coordinate the expression of traits that confer evolutionary advantages (1, 2). This “safety-in-numbers” strategy relies on the production and accumulation of small signaling molecules known as autoinducers, whose concentration increases as the population grows (3). Once a critical threshold concentration is reached, these signals trigger regulatory cascades that activate genes associated with collective behaviors, including biofilm formation, plasmid transfer, bioluminescence, and virulence factor production (4–6). These behaviors occur both intra- and interspecifically (7). Through the synthesis and detection of autoinducers, bacteria translate population density and community composition into synchronized, adaptive group behaviors (8).

*Acidithiobacillus ferrooxidans* and *Acidithiobacillus thiooxidans* are acidophilic, chemolithoautotrophic bacteria distinguished by their abilities to oxidize metal-sulfide minerals (9–11). While *A. ferrooxidans* can oxidize both ferrous iron and reduced sulfur species, *A. thiooxidans* only oxidizes reduced sulfur species (12). They are members of microbial consortia that can lead to acid mine drainage, and their metabolic abilities are exploited in industrial bioleaching operations (13). They can possess several quorum-sensing systems, including the *N-*acyl homoserine lactone (AHL), Autoinducer-2 (AI-2), diffusible signal factor, and bis-(3′-5′)-cyclic dimeric GMP (c-di-GMP) pathways (14). The AHL-mediated system is the most characterized and closely linked to biofilm formation (15). In this system, a synthase protein AfeI produces AHL autoinducers, which consist of a conserved homoserine lactone ring attached via an amide bond to a variable acyl side chain (typically C8–C16 in length) and frequently modified by oxo or hydroxyl substitutions (16, 17). As threshold concentrations are reached, AHLs bind to the receptor protein AfeR, activating the transcription of QS-regulated genes that influence metabolism, genetic regulation, and biofilm formation (18). QS-induced biofilm formation occurs through the onset of cell surface-mineral interactions and the production of capsular polysaccharides; however, the process is not fully characterized (19, 20).

Biofilm formation plays a critical role in industrial metal bioleaching, where the acidophilic microbial consortium facilitates metal extraction from ores through contact and non-contact mechanisms (21, 22). In the contact mechanism, mineral solubilization is mediated by biofilm formation directly on the mineral surface; thereby, bioleaching efficiency is strongly influenced by biofilm development (23). Biofilm can enhance metal recovery through improved surface contact for some ores or, conversely, inhibit leaching through the formation of interfacial microenvironments that promote the accumulation of passivation-forming species (24, 25). As global demand for critical materials and sustainable mining practices increases, a deeper understanding and precise control of QS dynamics in acidophilic microbial communities will be essential for optimizing bioleaching performance (26). *A. ferrooxidans* has recently been engineered to enhance bioleaching efficiency through the targeted manipulation of energy metabolism (25) and overexpression of quorum-sensing genes (27, 28). However, the impact of quorum quenching on microbial communities, biofilm formation, and overall bioleaching efficiency in these species remains understudied.

Quorum quenching is widely used to disrupt bacterial community communications by pathogens, such as *Pseudomonas aeruginosa*, *Vibrio cholerae, Vibrio harveyi,* and *Staphylococcus aureus* (29). In AHL-based QS systems, quorum quenching can be achieved through the use of inhibitory molecules, such as AHL analogs or AHL-degrading enzymes, which interfere with signal recognition by receptor proteins (30). Quorum-quenching enzymes are broadly classified into three categories: lactonases, acylases, and oxidoreductases (31). Lactonases inactivate AHL by hydrolyzing the ester bond of the homoserine lactone ring into a linearized acyl-homoserine molecule incapable of activating its cognate transcription factor (32, 33).

Most characterized AHL lactonases belong to the metallo-β-lactamase (MBL) family and contain a conserved dinuclear metal-binding motif (HXHXDH), in which histidine and aspartate side chains coordinate the catalytic metal ions (32, 34). AHL lactonases may function as endogenous regulatory mechanisms, enabling bacteria to self-attenuate QS under stress conditions, during plasmid transfer, and across growth phases when the transcription of QS genes may be disadvantageous or energetically unfavorable (35, 36). Additionally, the heterologous introduction of MBL-type lactonases into various microbiomes promoted the disassembly of biofilms (37–39).

In this study, we identify and characterize an AHL lactonase gene product from *A. ferrooxidans* strains TFBk and BY-3, designated AiiF, through bioinformatic analysis and biochemical characterization. We evaluate its kinetic properties and employ ligand docking simulations to examine the influence of AHL acyl chain length and substitution patterns on substrate binding affinity, providing insight into its catalytic mechanism. We demonstrate the AHL-degrading activity of AiiF and assess the impact of quorum quenching on biofilm formation in acidophiles. Finally, we transformed *A. thiooxidans* to recombinantly express AiiF and demonstrate the ability to modulate biomining-relevant phenotypes within bioleaching systems via quorum quenching.

## RESULTS

**Fig 1:**
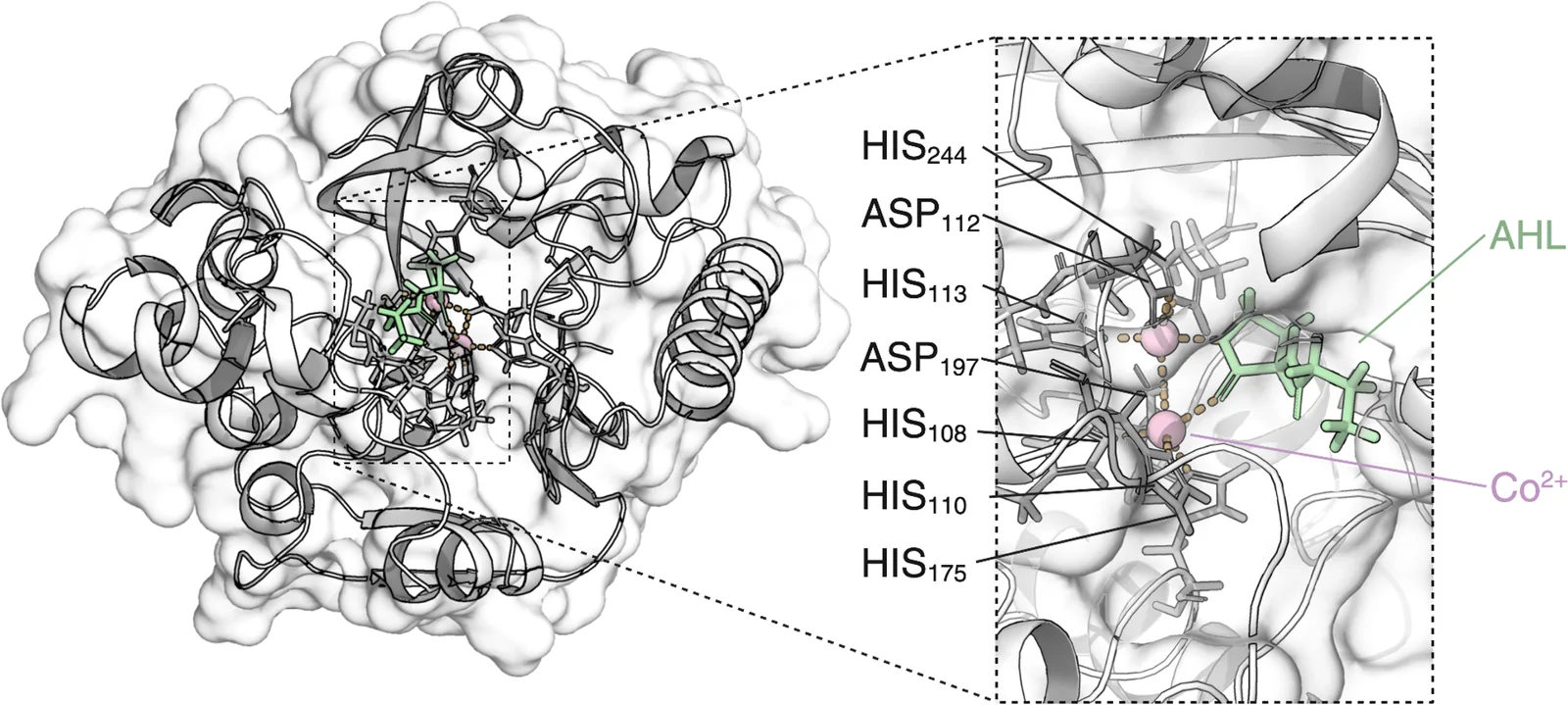
Structural model of AHL lactonase AiiF from *Acidithiobacillus ferrooxidans*. The overall protein fold (left), predicted using AlphaFold3, reveals an MBL-like architecture with an open catalytic cleft. The inset (right) highlights the active site containing the conserved metal-binding motif HXHXDH and coordinating residues (His108, His110, Asp112, His113, His175, and Asp197). Two cobalt ions are predicted to occupy the catalytic center, forming a binuclear metal site. An AHL substrate is shown docked within the catalytic pocket, positioned for hydrolysis by the metal-dependent mechanism.

A hypothetical lactonase protein native to *A. ferrooxidans* was identified through a bioinformatics search and designated AiiF (autoinducer inhibitor from *Acidithiobacillus ferrooxidans*). AiiF is predicted to be a 29.3 kDa protein exhibiting strong homology (E-value = 3 × 10^−34^) to the query protein AHL lactonase AiiA from *Bacillus thuringiensis* (40). BLASTp analysis confirmed the presence of the AiiF gene in both the TFBk (NZ_JANJYU010000072.1) and BY-3 (NZ_AZNR01000116.1) *A. ferrooxidans* genomes (41); however, it is not found in the genome of the commonly studied ATCC 23270 type strain. Sequence analysis revealed conservation of the canonical metal-binding motif, HXHXDH, leading to the predicted binding of two cobalt ions within the open catalytic cleft by AlphaFold3 (42). The predicted metal coordination geometry resembles that of AiiA, with both cobalt ions positioned to participate in the catalysis of AHL hydrolysis (Fig. 1).

**Fig 2:**
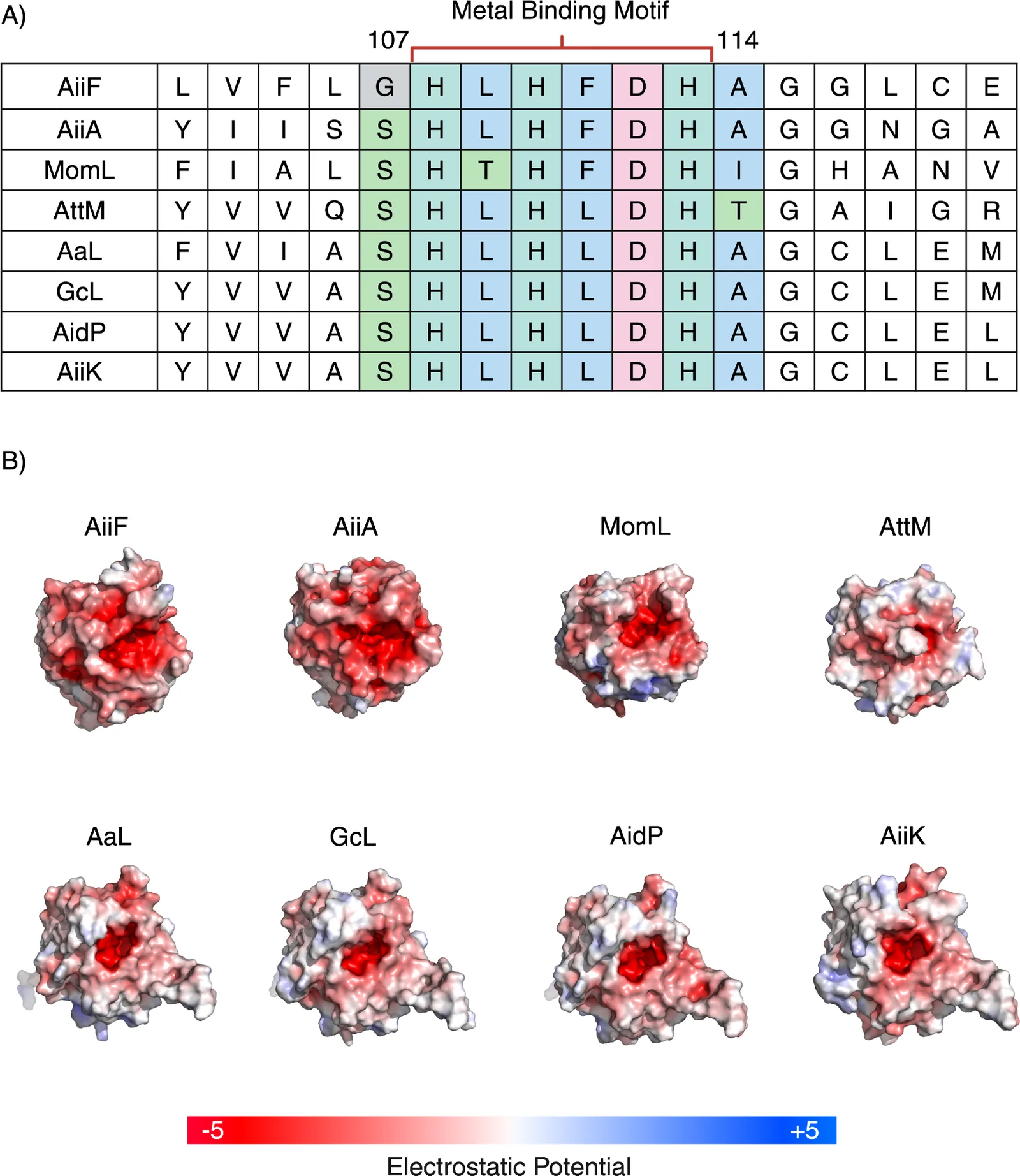
Sequence and structural comparison of AiiF with representative AHL lactonases from the MBL family. (**A**) Sequence alignment of AiiF with representative MBL-family AHL lactonases, highlighting the conserved metal-binding motif HXHXDH (residues 107–114 in AiiF). In AiiF, the motif is preceded by a glycine, whereas other MBLs contain a serine at this position, which may increase flexibility within the metal-binding pocket and influence metal ion preference. Amino acids are color-coded based on Clustal guidelines, where blue indicates hydrophobic, pink indicates negative charge, green indicates polar, gray indicates glycines, and teal indicates aromatic. (**B**) Predicted surface electrostatic potential maps of AiiF and representative MBL-family AHL lactonases. AiiF and AiiA display highly similar structures with strongly negatively charged binding clefts, whereas AaL, MomL, AttM, GcL, AidP, and AiiK exhibit clefts with significantly less surface charge. The polarity of the binding cleft is predicted to influence enzyme selectivity and catalytic activity. Full sequences and accession numbers are listed in Table S1.

AiiF is a member of the MBL family, as indicated by a protein sequence alignment with representative AHL lactonases and the presence of the conserved metal-binding motif HXHXDH (Fig. 2A), which is characteristic of MBLs (43). The binding motif in AiiF is preceded by a glycine, distinct from all other selected MBLs, which contain polar serines at this position (Fig. 2A). Glycine substitutions could increase the structural flexibility of the binding pocket and contribute to metal ion preferences, which are known to be highly variable among MBLs (44). Although MBLs exhibit varying substrate preferences and catalytic mechanisms, sequence similarity is observed across MBLs relative to other families, including α/β-hydrolase fold (AidH), phosphotriesterase-like lactonase (SsoPOX), and paraoxonase (PON2) (Fig. S1). In order from closest to most distant sequence similarity to AiiF, the lactonases are ranked: AiiA, AiiB, AaL, GcL, AidP, AiiK, AttM, MomL, AidH, SsoPOX, and PON2 (Fig. S2) (37, 38, 40, 45–52).

AiiF was the only MBL-like protein detected in the available genomes of *A. ferrooxidans* strains and exhibited the greatest sequence homology to AiiA. The high sequence homology between AiiF and AiiA results in similar predicted structures compared to other MBLs (Fig. 2B). The AHL-binding clefts of both AiiF and AiiA are predicted to be lined with negatively charged amino acids (indicated in red). Conversely, the AHL-binding clefts of AaL, MomL, AttM, GcL, AidP, and AiiK are predicted to be lined with amino acids carrying less surface charge (indicated in white). The polarity of the binding cleft affects both the selectivity and activity of the enzyme. Of these structures, AaL, AttM, GcL, and AiiK appear highly similar, consistent with the phylogenetic tree (Fig. S2).

**Fig 3:**
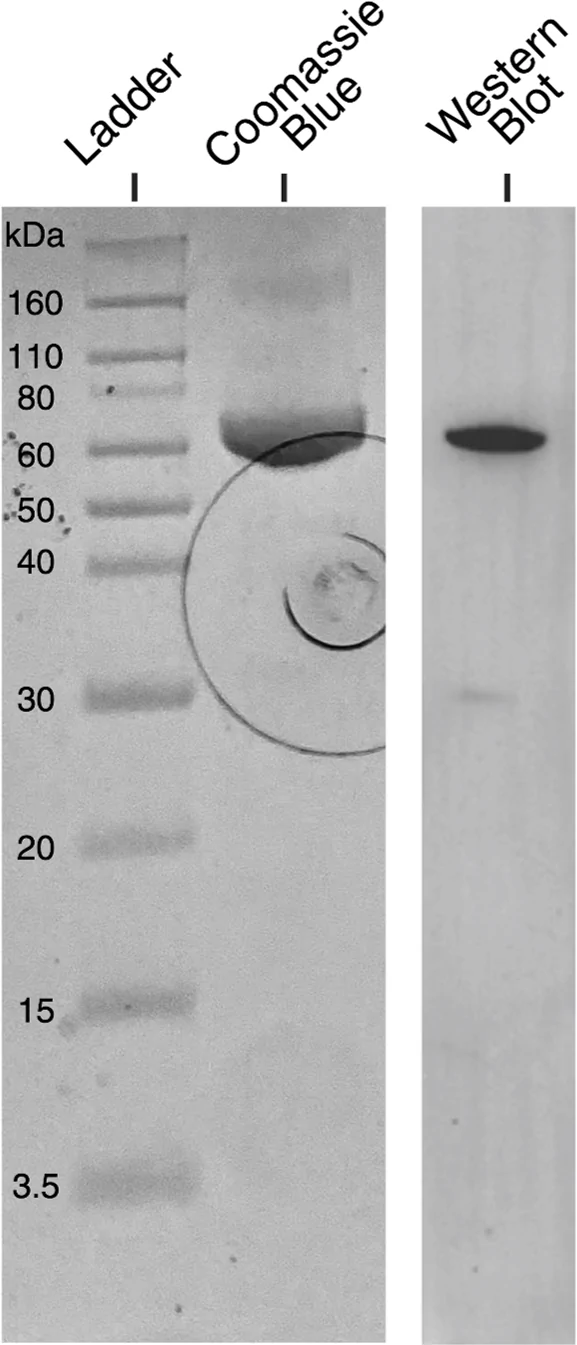
Expression and purification of MBP-AiiF fusion protein. A Coomassie Blue-stained SDS-PAGE gel shows a prominent band in the 60–80 kDa range, indicating successful purification with a purity greater than 95%. A western blot probed with a C-terminal Strep-tag II antibody confirms the identity of the purified protein. The molecular weight ladder is shown in the first lane with marker sizes indicated in kDa.

Tag-free overexpression of AiiF in *Escherichia coli* resulted in low yields, inclusion body formation, and poor solubility. Therefore, AiiF was expressed and purified as a fusion with the maltose-binding protein (MBP). The 73 kDa fusion protein was purified using an amylose resin system, yielding soluble protein with purity exceeding 95% as determined by SDS-PAGE, and its identity was confirmed by western blotting using a C-terminal Strep-tag II (Fig. 3). Although MBP is a relatively large protein (42.6 kDa), previous studies have shown that MBP fusions do not affect the activity of other lactonases (53).

**Fig 4:**
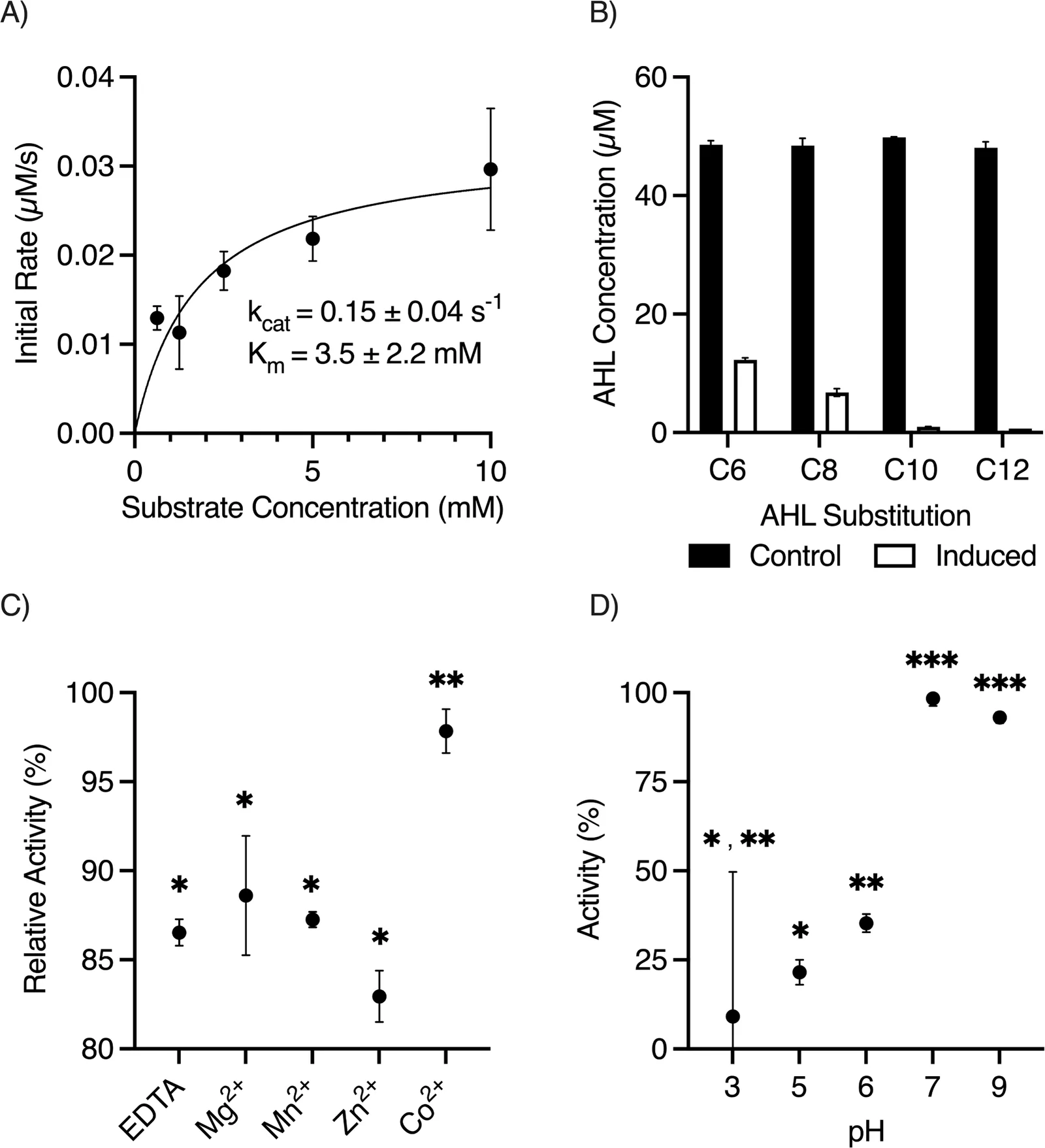
Evaluation of AiiF AHL-degrading kinetics and selectivity. (**A**) An *in vitro* phenol red assay was used to measure initial rates. A Michaelis-Menten curve was fitted to the initial rates of the degradation of C4-HSL by purified MBP-AiiF enzyme. All initial rates were measured in triplicate, and the error estimates of the kinetic parameters represent a 95% confidence interval of the fit. (**B**) An *in vivo* biosensor luminescence assay in *E. coli* was used to test the degradation of AHL substrates. Biosensor pSB1142 was used for long-chain AHLs (C10- and C12-HSL); biosensor pSB401 was used for medium-chain AHLs (C6- and C8-HSL). AiiF expression was induced with the addition of IPTG, and the control was not induced. Biosensor assays were performed in biological triplicate, and error bars on both graphs represent standard error. (**C**) Relative activities of purified MBP-AiiF were determined using phenol red kinetic assays supplemented with varying metal ions to obtain initial rates. Relative activities were calculated with respect to the fastest rate, obtained with cobalt. (**D**) Activity as a function of pH represents the amount of residual C4-HSL after *in vitro* reactions of AiiF-MBP compared to the starting amount in buffers with varied pH, and AHL concentration was measured using the AHL biosensor plasmid pSB536 harbored in *E. coli*. Raw luminescence values were normalized with OD600 values, and the resulting curve was integrated over time to obtain normalized luminescence values. Activities were calculated as the amount of quenched normalized luminescence relative to starting normalized luminescence values. Error bars were calculated for both panels C and D using the standard error of the mean with ordinary one-way ANOVA and Brown-Forsythe and Welch ANOVA tests, respectively. *, **, and *** represent groups that are distinguished by *P* < 0.05. All experiments were performed in triplicate. Figures and analyses were generated using Prism 10.

The hydrolysis of AHL by AiiF releases a proton, increasing acidity and enabling the use of a pH-indicating dye (phenol red) to monitor reaction progress *in vitro* (50, 54). Initial rates of C4-HSL degradation at varying starting substrate concentrations were measured over 30 min and were fit by the Michaelis-Menten equation (Fig. 4A). The estimated kinetic parameters for C4-HSL degradation by AiiF yielded a *k*_cat_ of 0.15 ± 0.04 s^−1^ and a *K*_m_ of 3.5 ± 2.2 mM. The kinetics of AiiF are relatively slow compared to other MBL family members reported in the literature (Table S2). Due to the poor solubility of longer-chain AHLs and the relatively high *K*_m_ of AiiF, the *in vitro* assay was limited to C4-HSL due to its higher solubility.

Alternatively, an *in vivo E. coli*-based assay was used to evaluate the enzyme selectivity and its ability to degrade medium-chain (C6- and C8-HSL) and long-chain (C10- and C12-HSL) AHLs (Fig. 4B). Cells harboring AHL-responsive biosensor circuits with luminescence output and encoding AiiF under an inducible promoter were used to detect AiiF-mediated degradation of AHLs. Luminescence signals produced in response to the different AHL molecules were quenched upon induction of AiiF expression. AHL-dependent luminescence was reduced for all tested chain lengths, supporting AiiF as a broad-range lactonase.

The biosensor circuits differed between substrates due to chain-length specificity; therefore, relative activity comparisons could not be made. However, these results demonstrate that AiiF degrades C6-, C8-, C10-, and C12-HSL *in vivo*. Activity was observed at AHL concentrations of 50 μM, which is well below the measured *K*_m_ value. This reflects differences between *in vitro* and *in vivo* conditions, including enzyme localization, local intracellular AHL concentrations, and the higher sensitivity of the biosensor assay compared to the pH-based assay, which enabled testing of longer-chain AHLs at lower concentrations *in vivo*.

The enzymatic activity of AiiF as a function of various metal ions and pH conditions was also evaluated (Fig. 4C and D). Cobalt-bound AiiF exhibited the highest relative activity; however, the enzyme retained most of its function in the absence of metal ions (EDTA condition). In the presence of magnesium, manganese, or zinc, relative activity did not differ significantly from the EDTA condition, highlighting the unique activity with cobalt. The effect of pH on activity can be rationalized by protonation of key catalytic side chains (pK_a_ of histidine ~6). Consistent with this, AiiF exhibited maximal activity at pH 7, near the reported intracellular pH of *A. ferrooxidans* (10), with a significant decrease observed at pH 6. Nevertheless, enzymatic activity remained detectable at pH 3.

Docking analyses suggest that AiiF binds short-chain AHLs more strongly than long-chain AHLs (Table S3). Glide scores, which approximate ligand binding energies, become less negative with increasing chain length from C4- to C10-HSL, indicating weaker predicted binding. This trend was observed for unsubstituted HSLs, 3-oxo-HSLs, and 3-hydroxy-HSLs. Conversely, in all cases, the predicted binding energy of C14-HSL is more negative than that of C12-HSL, stemming from increased acyl chain stabilization in C14.

Substituted AHLs (3-hydroxy-HSLs and 3-oxo-HSLs) exhibited lower binding energies overall compared to unsubstituted HSLs. This trend was also examined experimentally for C4-HSL, where substituted HSLs showed higher apparent reaction rates than the unsubstituted form (Fig. S3). However, ordinary one-way ANOVA with Tukey’s multiple comparisons test indicated that the observed rate constants did not differ significantly (*P* > 0.05).

Ligand interaction diagrams of AHLs docked into AiiF reveal that acyl chain stabilization and metal coordination vary with chain length (Fig. S4 through S6). Specifically, longer-chain substrates exhibit greater solvent exposure of the acyl tail and reduced stabilization by hydrophobic interactions. In addition, the carboxyl group of Asp112, proposed to participate in the catalytic mechanism, shifts farther from the lactone carbonyl group as chain length increases. Among C4-HSL derivatives, the 3-hydroxy substitution displays the strongest predicted acyl chain stabilization, including a hydrogen bond between the hydroxyl group and Phe74.

**Fig 5:**
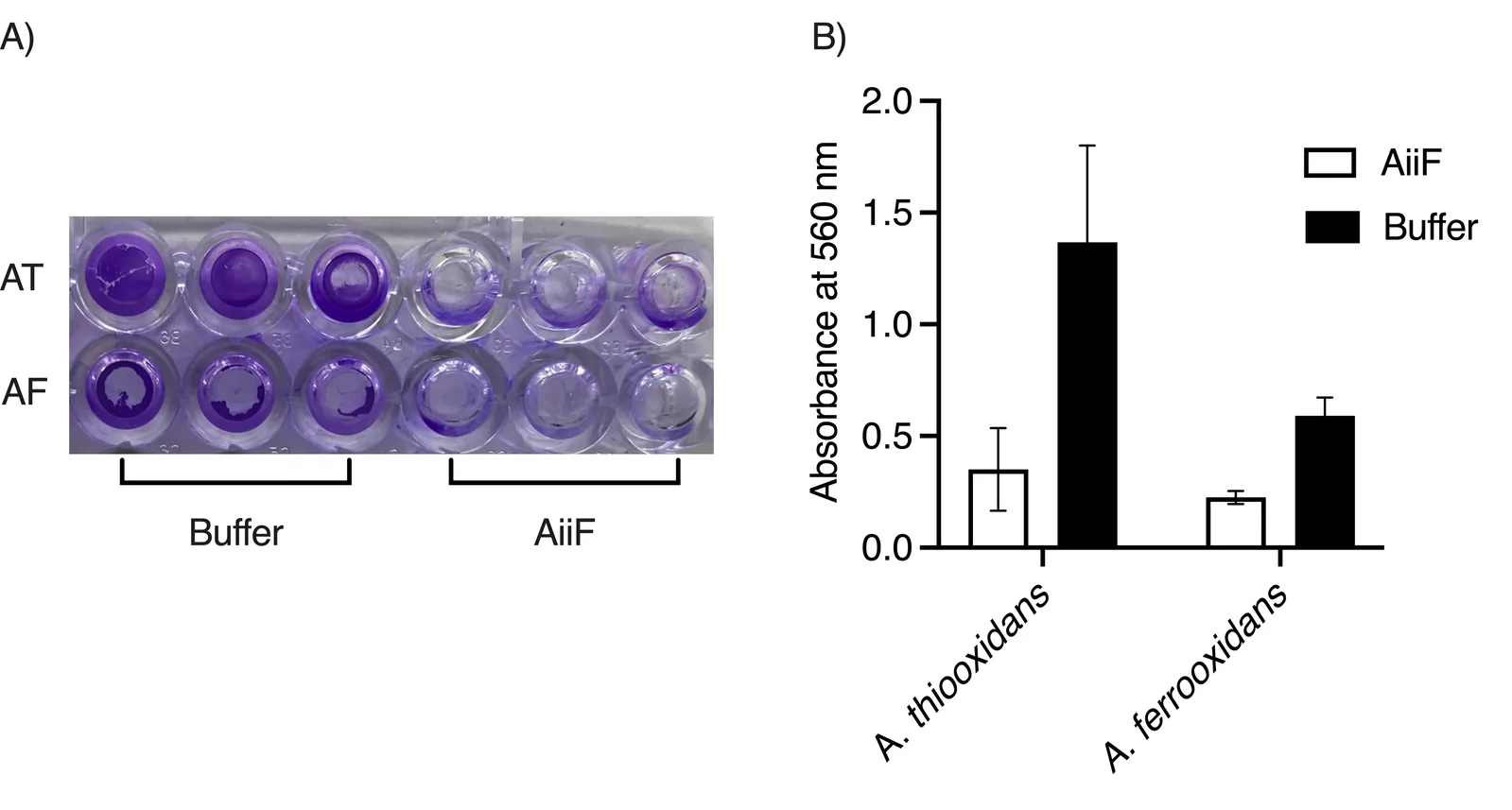
Effect of exogenous AiiF on biofilm formation in *Acidithiobacillus* species. (**A**) Crystal violet-stained biofilms formed by *A. thiooxidans* (ATCC 19377) and *A. ferrooxidans* (ATCC 23270) grown in sulfur medium (initial pH 5) in 96-well plates. Cultures supplemented with purified AiiF showed visibly reduced biofilm formation compared with buffer controls. (**B**) Quantification of biofilm biomass by measuring the absorbance of solubilized crystal violet at 560 nm. Cultures treated with AiiF exhibited reduced absorbance relative to buffer controls, indicating decreased biofilm formation. Data represent the mean of three biological replicates with standard error of the mean.

The type strains *A. ferrooxidans* (ATCC 23270) and *A. thiooxidans* (ATCC 19377) can both grow via oxidation of dispersed sulfur, a medium that supports a relatively high initial pH (pH 5) and directly influences quorum-sensing gene regulation, including biofilm formation (25). To evaluate the effect of AiiF on biofilm development, purified enzyme was added exogenously to cultures of both species growing in sulfur media at daily intervals to amplify its impact in the harsh extracellular acidic conditions prior to staining biofilms with crystal violet (55). Crystal violet-stained biofilms formed in 96-well plates were visibly reduced in cultures supplemented with AiiF compared to buffer controls (Fig. 5A). *A. thiooxidans* formed more developed biofilm than *A. ferrooxidans* under these conditions; however, biofilm reduction was evident in both species. Quantification of solubilized crystal violet confirmed the visual observations, with lower absorbance values measured in wells receiving AiiF supplementation (Fig. 5B). AiiF lacks a predicted secretion signal and is therefore not expected to be secreted by or exist in the extracellular environment of these organisms; thus, these experiments were performed as a proof-of-concept prior to engineering these non-model organisms.

**Fig 6:**
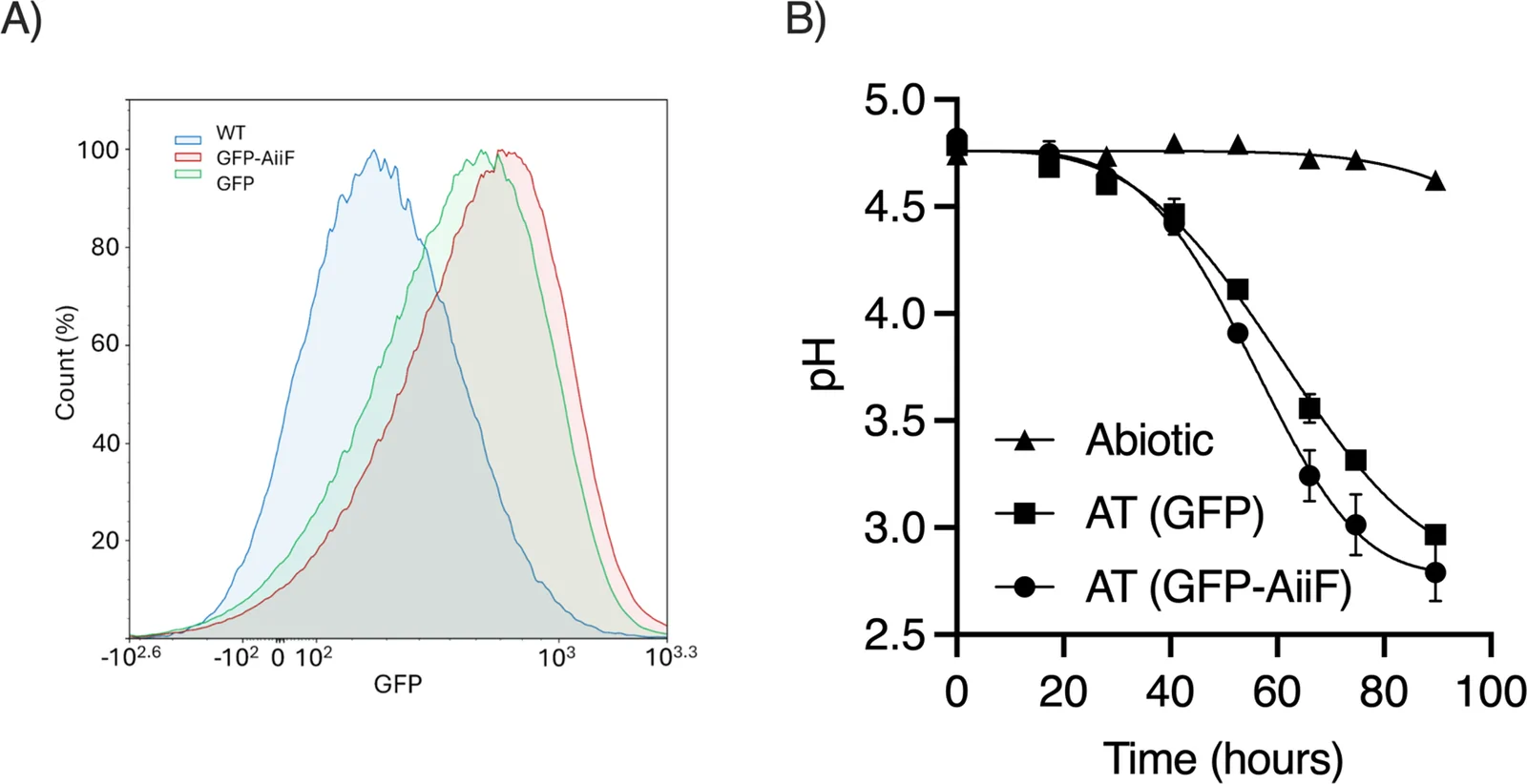
AiiF expression and effect on the growth of *Acidithiobacillus thiooxidans*. (**A**) GFP fluorescence shift in *A. thiooxidans* expressing GFP and GFP-AiiF compared to wild-type cells (WT). Overlaid histograms of normalized cell-count distributions show a clear rightward shift in fluorescence intensity, indicating an increased proportion of cells exhibiting GFP expression in GFP and GFP-AiiF relative to WT. (**B**) Growth of engineered *A. thiooxidans* strains monitored by pH decline over time, which reflects sulfur oxidation and cellular growth. The GFP-AiiF expressing strain (circles) exhibited a more rapid decrease in pH compared to the GFP control (squares), indicating accelerated growth. An abiotic control (triangles) showed minimal pH change. *A. thiooxidans* growth is correlated to pH decline, and this is fit to the Weibull decay model (solid black line). Samples were grown in triplicate, and error bars represent standard deviation.

The AiiF gene was cloned into a pJRD215 plasmid and fused to superfolder green fluorescent protein (GFP) at the N-terminus under a constitutive tac promoter using primers in Table S4 (56, 57). Expression plasmids containing GFP and the GFP-AiiF fusion were electroporated into *A. thiooxidans,* and transformations were confirmed by colony PCR (Fig. S7). GFP expression was evaluated by flow cytometry (Fig. 6A), and both the GFP and GFP-AiiF expressing cells were similarly fluorescent, indicating intracellular expression of the transgenes in *A. thiooxidans*. Surprisingly, growth of the engineered strains was affected by the expression of AiiF, as indicated by a more rapid decrease in medium pH, which correlates with *A. thiooxidans* growth (58) (Fig. 6B). The GFP-AiiF expressing strain exhibited accelerated growth relative to the control, as quantified by fitting to the Weibull decay model, in which the scale parameter *k* describes the rate of change (59, 60). The lactonase-expressing strain displayed a higher growth rate (*k*_AiiF_ = 0.016 h^−1^, 95% CI 0.016 to 0.017) compared to the GFP control strain (*k*_GFP_ = 0.015 h^−1^, 95% CI 0.014 to 0.015), with the difference being statistically significant (*P* = 0.002). In contrast, the shape parameter, *n*, did not differ significantly between strains (*P* = 0.08), with values of *n*_AiiF_ = 4.0 ± 0.6 and *n*_GFP_ = 3.3 ± 0.4.

**Fig 7:**
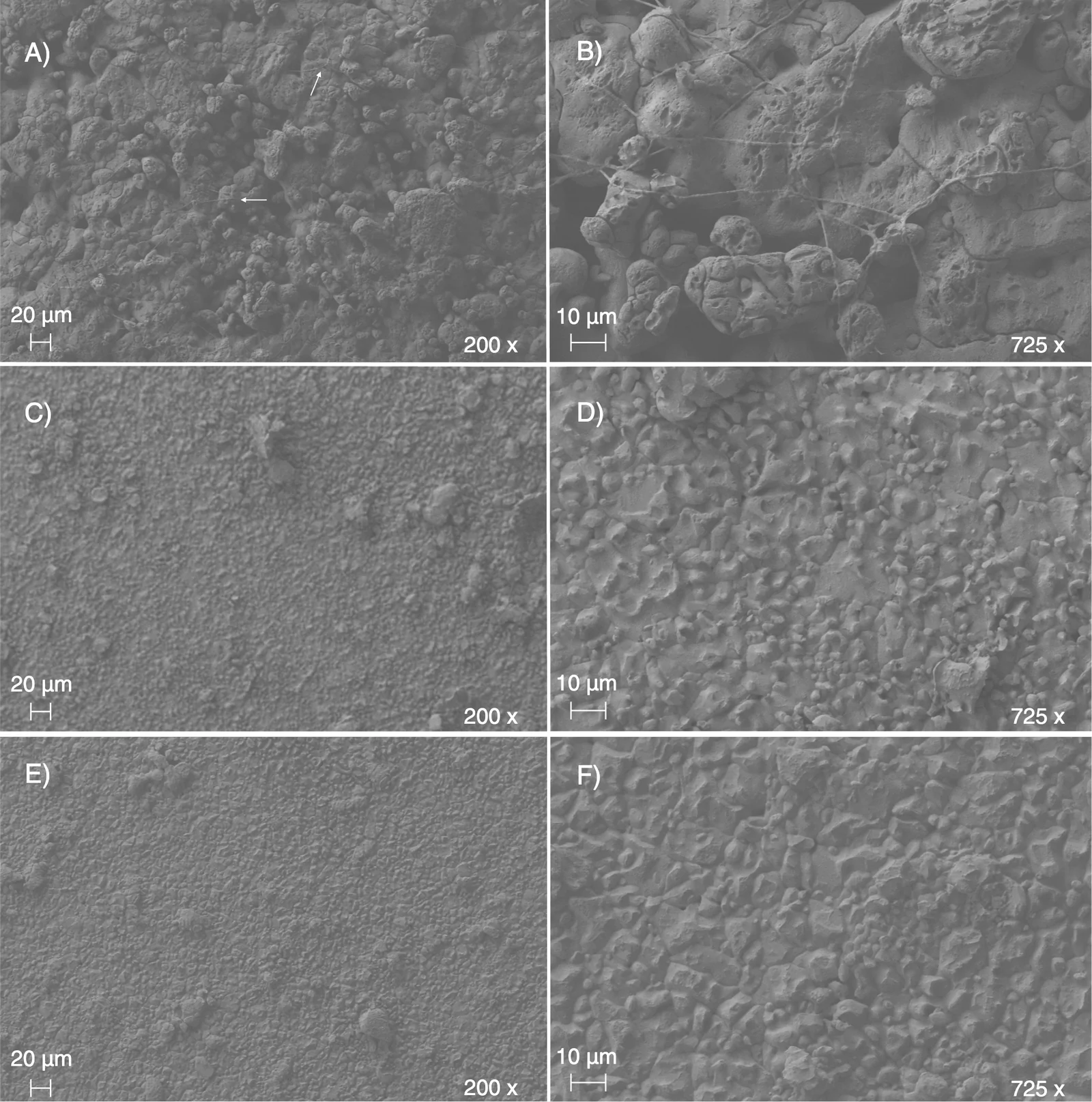
Scanning electron microscopy analysis of sulfur coupon surfaces colonized by engineered *A. thiooxidans* strains. (**A and B**) Coupons inoculated with the GFP control strain exhibit pronounced surface pitting and cavity formation, and abundant EPS is visible across the surface. (**C and D**) Coupons colonized by the GFP-AiiF expressing strain show markedly reduced EPS deposition and limited surface alteration. (**E and F**) Abiotic control coupons display surface morphologies comparable to those observed in the lactonase condition. Scale bars: 20 μm in panels A, C, and E (200× magnification); 10 μm in panels B, D, and F (725× magnification). Representative images from three independent replicates (*n* = 3).

Surface colonization and biofilm development of engineered *A. thiooxidans* strains on sulfur coupons, simulating bioleaching conditions, were examined by scanning electron microscopy (SEM). Notable differences in surface morphology were observed between coupons colonized by the GFP-AiiF expressing strain and those colonized by the GFP control (Fig. 7). Coupons inoculated with the GFP control strain exhibited extensive surface pitting and cavity formation, consistent with enhanced sulfur dissolution (Fig. 7A and B). In addition, abundant extracellular polymeric substances (EPS) were observed across the surface, with accumulations particularly evident within surface crevices. In contrast, coupons colonized by the GFP-AiiF expressing strain displayed minimal EPS deposition and substantially reduced surface alteration (Fig. 7C and D). The overall morphology of the GFP-AiiF coupons closely resembled that of the abiotic control (Fig. 7E and F). Although small, localized EPS deposits were occasionally observed, their presence was evidently reduced compared to the GFP controls.

## DISCUSSION

In this study, an AHL lactonase native to two *A. ferrooxidans* strains was identified and characterized. A phylogenetic analysis revealed that AiiF shares the highest sequence similarity with AiiA from *B. thuringiensis* (Fig. 2A and B). The two enzymes exhibit similar kinetic behavior, with reported *K*_m_ values for C4-HSL of 3.5 mM for AiiF (Fig. 4A) and 5.1 mM for AiiA (33). These values are notably higher than those reported for several phylogenetically distant MBLs (Table S2). Previous studies have shown that the hydrophobicity and size of residues lining the substrate-binding pocket can strongly influence substrate promiscuity and catalytic activity in quorum-quenching enzymes (61). Several MBL lactonases with distinctive non-polar clefts lining their binding pockets, including MomL, AttM, AaL, GcL, AidP, and AiiK, display increased activity toward AHL molecules, which possess bulky hydrophobic acyl chains (62). These hydrophobic binding clefts likely assist in orienting the non-polar chains of AHL molecules within the active site. In contrast, AiiA and AiiF contain more charged amino acids and a higher surface charge around the binding site, which likely contributes to their comparatively higher *K*_m_ values.

The HXHXDH metal binding motif is highly conserved among MBLs (49, 63, 64). However, metal ion preference and metal dependence are extremely diverse across different species (65–67). A study of AHL degradation by AiiA showed that the di-zinc motif is essential for coordinating the bridging hydroxide ion, which can form a hydrogen bond with a localized aspartic acid and assist in facilitating the nucleophilic attack on AHL (32). Conversely, an enzymatic study of AiiK showed that AHL-degrading activity persisted after chelator treatment, with the addition of metal ions resulting in either inhibitory or enhancing effects (38). These differences can be attributed to numerous factors, including the mechanistic promiscuity of MBLs, which has been shown to alternate between at least two different mechanisms: stepwise nucleophilic attack by a terminal hydroxide ion and stepwise base-catalyzed attack via metal-coordinated aspartic acids (61). Furthermore, metal ion supplementation during protein expression can strongly impact kinetic parameters and apparent activity (49). All of these factors contribute to the metal ion preferences of AiiF exhibited in Fig. 4C, which, like AiiK, shows variable activity across metal ions and retained activity in the absence of metal ions. Interestingly, this preference contrasts with the proposed metal dependency of its closest relative, AiiA (34).

Binding affinity to AiiF decreases with increasing acyl chain length for unsubstituted, 3-oxo-, and 3-hydroxy-HSLs, as predicted by GlideScore docking (Table S3). Ligand binding diagrams indicate that short-chain AHLs are stabilized by amino acids lining the active site, allowing hydrophobic interactions to be maintained along the acyl chain. In contrast, longer acyl chains are more solvent-exposed and form weaker interactions. This trend persisted through C12, with C14 showing increased binding affinity relative to C12. The C14 acyl chain appears more stabilized than C12 and, in some cases, exhibits increased metal coordination. The predicted binding affinities of hydroxyl-HSLs were higher than unsubstituted, which may result from hydrogen bonding between the hydroxyl group and phenylalanine residues or other hydroxyl-mediated interactions that stabilize the acyl chain. Binding energy has previously been shown to correlate linearly with AHL-degrading activity for AiiA, suggesting that docking-derived binding energies may approximate activity trends (68–71). These predictions, therefore, indicate that AiiF may exhibit increased activity toward short-chain and substituted HSLs. Notably, short-chain HSLs are uncommon in the native environment of *A. ferrooxidans*, which primarily synthesizes medium- and long-chain HSLs (16, 17). It is possible that AiiF may target AHL molecules produced by other members of the consortia to interfere with competing quorum-sensing activities. While these docking results provide insight into how ligand interactions vary across substrates, further biochemical validation is required.

Due to the poor solubility of AHL substrates, *in vitro* assays were limited to C4-HSL. The observed rate constants for unsubstituted, hydroxyl, and oxo-C4-HSLs were 1.5 ± 0.5, 2.0 ± 0.9, and 2.5 ± 0.2 s^−1^, respectively (Fig. S3), supporting the modeling prediction that substituted HSLs exhibit higher catalytic activity by AiiF compared to unsubstituted HSLs. These values, however, were not statistically different (*P* > 0.05), which may be attributed to the relatively small differences in predicted binding energies (−4.07, −4.22, and −4.13 for unsubstituted-, hydroxyl-, and oxo-C4-HSL, respectively). We predict that AHLs with greater differences in predicted binding energies, such as −4.07 for C4-HSL and −0.569 for C12-HSL, would exhibit more pronounced differences in kinetic behaviors.

In addition to predicting substrate specificity, ligand-protein diagrams provide insight into the AHL degradation mechanism of AiiF (Fig. S4 through S6). Several proposed mechanisms for MBL lactonases suggest that metal ions coordinate oxygen atoms of the substrate, promoting a nucleophilic attack. Consistent with these models, metal-oxygen coordination is observed in the AiiF ligand-protein diagrams in most cases. Asp112 is located near the catalytic active site and may contribute to nucleophilic activation. Variation in the positioning of the aspartate side chain and the substrate carbonyl relative to the cobalt ions further supports theories of mechanistic promiscuity in AHL lactonases, suggesting that AiiF may proceed through multiple catalytic mechanisms in a substrate-dependent manner.

The presence of an endogenous AHL lactonase within the genomes of organisms that inhabit microbial consortia known to use cell-cell signaling warrants consideration, as this suggests a role for quorum-sensing attenuation. For instance, *Agrobacterium tumefaciens* utilizes native QS pathways but also harbors the AHL lactonase AttM, which is upregulated in the stationary phase and after conjugal transfer (35). Similarly, second-messenger pathways, such as c-di-GMP, modulate biofilm production in response to nutrient availability (72, 73), and this response is speculated to minimize the high-energy impact associated with biofilm formation under low nutrient conditions (74). BY-3 strains are known to be AHL-responsive, and both BY-3 and TFBk possess putative LuxI homologs (75, 76). By analogy, we propose that these *A. ferrooxidans* strains likely utilize AiiF to modulate resource allocation when further investments in biofilm production or other QS traits are no longer advantageous. Furthermore, *A. ferrooxidans* is known to live within consortia that contain members capable of responding to AHL molecules, such as *Acidithiobacillus thiooxidans* and *Leptospirillum ferrooxidans* (75, 77), and these relationships are known to be agonistic or cooperative depending on the environmental context. However, in most reported cases, overall iron oxidation is improved by mixed communities (78, 79). Therefore, AiiF may provide *A. ferrooxidans* with a mechanism to control communal outputs and coordinate beneficial group behaviors. Future temporal studies of AiiF expression in response to QS molecules, growth phase, and consortia will be valuable for clarifying when this modulation occurs and to better understand communal functionality within biomining systems.

This study demonstrates that AHL lactonases can be employed to effectively disrupt biofilm formation within bioleaching cultures. Previous studies have shown the direct relationship between the addition of exogenous AHL and biofilm production in *A. ferrooxidans* (15). In agreement with these studies, we show that reducing the AHL concentration via extracellular enzymatic degradation reduces the biofilm content for both *A. ferrooxidans* ATCC 23270 (which does not have a homologous AiiF gene) and the sulfur-oxidizing *A. thiooxidans* (Fig. 5). This was accomplished by adding AiiF to the low pH growth media, where the enzyme is inhibited but still active (Fig. 4D).

The acidophiles maintain a circumneutral intracellular pH, and we recombinantly expressed the *A. ferrooxidans* AiiF in *A. thiooxidans*. As the enzyme was expressed as a fusion to GFP, the green cells indicate intracellular expression of the AiiF gene (Fig. 6A). The expression of AiiF increased cell growth rates (Fig. 6B). We hypothesize that this behavior is due to the observed reduction in biofilm formation, which could alter resource allocations or cell-surface interactions; however, this would need verification in future studies. The intracellular expression of AiiF dramatically reduced EPS deposition and surface colonization on sulfur coupons, indicating that enzymatic quorum quenching can regulate biofilm formation and surface morphology of the *A. thiooxidans* cells when grown on a sulfur substrate (Fig. 7).

Although biofilm formation is often thought to correlate with increased bioleaching efficiency, recent advances show that mineral composition and surface reactions are involved in determining the overall efficiency (25). The bioleaching of some complex polysulfides like chalcopyrite can be inhibited by biofilm formation, and this can be due to surface passivation by the formation of insoluble sulfur compounds (80, 81). Therefore, AHL lactonases, such as AiiF, may be useful for modulating biofilm formation, which may be leveraged to enhance bioleaching efficiencies of passivation-prone minerals.

## MATERIALS AND METHODS

### Chemicals and reagents

All chemicals were purchased from Sigma-Aldrich (St. Louis, MO, USA), unless specified otherwise.

### Bioinformatics search and plasmid construction

A bioinformatics search was conducted to evaluate sequence similarity between the quorum-quenching enzyme AiiA (UniProt ID P0CJ63) and predicted proteins from available *Acidithiobacillus ferrooxidans* genome sequences. Protein-protein alignments were performed using BLASTp. Statistical significance was assessed using the expected value (E-value), and sequences with E-values <1 × 10^−6^ were considered significant (41). The identified protein, hereafter designated AiiF, was codon optimized for expression in *Escherichia coli* using the Integrated DNA Technologies Codon Optimization Tool and synthesized as a gBlock gene fragment (Integrated DNA Technologies, Coralville, IA, USA). The optimized gene was cloned into the pMAL expression vector (82), generating an N-terminal fusion to MBP and a C-terminal Strep-tag II. DNA assembly reagents were obtained from New England Biolabs (Ipswich, MA, USA). Assembly was performed using the NEBuilder HiFi DNA Assembly Cloning Kit according to the manufacturer’s instructions. PCR primers were designed using the NEBuilder Assembly Tool (New England Biolabs) and are listed in Table S4. The resulting construct, pMal-AiiF, was transformed into chemically competent *E*. *coli* BL21(DE3) cells (New England Biolabs, Cat No. C2527H) and stored at −80°C for subsequent experiments. All DNA oligonucleotides and gene fragments were purchased from Integrated DNA Technologies (Coralville, IA, USA).

### Protein expression and purification

An overnight culture of *Escherichia coli* harboring the pMAL-AiiF vector was inoculated 1:100 into Terrific Broth and grown at 37°C to mid-log phase (OD₆₀₀ = 0.4–0.6). Protein expression was induced with 0.3 mM IPTG and 500 µM CoCl₂, and cultures were incubated at 30°C for 5 h. Terrific Broth was purchased from Research Products International (Mt. Prospect, IL, USA). IPTG and ampicillin were obtained from GoldBio (St. Louis, MO, USA). Cells were harvested by centrifugation at 4,700 × *g* for 15 min. Pellets were resuspended in modified maltose-binding protein wash buffer (20 mM Tris, 200 mM NaCl, pH 7.4) and lysed by sonication. Cell debris was removed by centrifugation at 10,000 × *g* for 1 h at 4°C, and the soluble fraction was filtered through a 0.22 µm PVDF syringe filter (CELLTREAT, Ayer, MA, USA). The clarified lysate was loaded onto an MBPTrap HP amylose column (Cytiva, Marlborough, MA, USA) using an ÄKTA pure system. The column was washed with 20 column volumes (100 mL) of wash buffer, and bound protein was eluted with 10 mM maltose. Elution fractions were pooled and concentrated using 50 kDa Amicon Ultra centrifugal filters. Protein purity was assessed by SDS-PAGE (Invitrogen, Waltham, MA, USA). The purified protein was desalted into reaction buffer (0.5 mM HEPES, 500 mM NaCl, pH 7.4) using PD-10 desalting columns (Cytiva, Marlborough, MA, USA). Western blot analysis of the MBP-AiiF-Strep-tag II fusion protein was performed according to the manufacturer’s instructions using monoclonal mouse primary antibodies and goat anti-mouse secondary antibodies. Primary antibodies (Invitrogen, MA5-37747) were used at a 1:4,000 dilution, and secondary antibodies (Bio-Rad, Immun-Star GAM-HRP conjugate, #1705047) were used at a 1:10,000 dilution. Approximately 20 µg of protein was loaded per lane. Protein concentration was determined using the Beer–Lambert law (83). The extinction coefficient was calculated using the ProtParam tool (84), and absorbance at 280 nm was measured with a UV-visible spectrophotometer.

### Sequence alignments and protein visualizations

All protein sequences were retrieved from the NIH GenBank database and are listed in the supplemental material. Multiple sequence alignments were performed using MEGA12 software with the MUSCLE algorithm (85). Alignments were further analyzed and visualized using Jalview v2.11.4.1 (86). Coloring schemes for figure generation were applied using the ClustalX color scheme. Phylogenetic analysis was conducted in Jalview using the BLOSUM62 substitution matrix. Electrostatic interactions were visualized in PyMOL using protein structure models predicted by AlphaFold3 (87, 88).

### *In vivo *lactonase activity biosensor assay

AHL-responsive bacterial biosensors were used to assess the AHL-degrading activity of AiiF *in vivo*. Plasmids encoding the biosensor components, pSB1142 and pSB401, were generously provided by Dr. Paul Williams (89). Each plasmid was individually co-transformed with the MBP-AiiF expression vector into chemically competent *Escherichia coli* 10-beta cells (New England Biolabs, Cat. No. C3019H; Ipswich, MA, USA). Overnight cultures of transformed cells were inoculated 1:100 into LB broth supplemented with the appropriate antibiotics and AHL and grown with shaking at 37°C to mid-log phase (OD₆₀₀ = 0.4–0.6). At this point, 200 µL of each culture was transferred into white, clear-bottom 96-well plates (Item No. 655098; Greiner Bio-One, Sattledt, Austria). Wells were supplemented with 0.3 mM IPTG or an equivalent volume of sterile deionized (DI) water. Plates were incubated at 30°C inside a microplate reader for 5 h, during which luminescence and absorbance at 600 nm were measured every 10 min (90). C10- and C12-AHL were classified as long-chain AHLs and were added to cells harboring pSB1142, whereas C6- and C8-AHL were classified as medium-chain AHLs and were added to cells harboring pSB401. AHL standards (C4-HSL, C6-HSL, C8-HSL, and C12-HSL) were purchased from Cayman Chemical (Ann Arbor, MI, USA) and dissolved in dimethyl sulfoxide (DMSO) to a stock concentration of 50 mM. Stocks were stored at −4°C until use. AHLs were diluted from 50 mM DMSO stocks into cell cultures to a final concentration of 50 µM. Luminescence values were normalized to OD₆₀₀, and normalized luminescence-time curves were integrated to determine area-under-the-curve values using the MATLAB trapz function (MathWorks, Natick, MA, USA). Calibration curves were generated for each AHL molecule and used to convert normalized luminescence values to approximate AHL concentrations.

### *In vitro* phenol red kinetic assay

A pH-based activity assay was used to determine the kinetic parameters for enzymatic degradation of AHL by AiiF, modified from previously described methods (54, 91, 92). Briefly, the reaction mixture consisted of a weakly buffered solution containing 0.5 mM HEPES and 500 mM NaCl (pH 7.4), supplemented with 200 µM CoCl₂ and C4-HSL. Water-soluble C4-HSL was directly dissolved in the buffered solution and serially diluted to final concentrations ranging from 10 to 0.125 mM using twofold dilutions. Phenol red, a pH-indicating dye, was added to achieve a final absorbance at 560 nm of approximately 1 (corresponding to ~0.0012%). All measurements were performed in cuvettes using a QuickDrop spectrophotometer (Molecular Devices, San Jose, CA, USA), which recorded absorbance at 560 nm every 5 s over a 30-min period. Time zero (*t* = 0) was defined as 5 s after the addition of enzyme to a final concentration of 250 nM. HCl titrations were conducted to determine the change in absorbance corresponding to proton generation. The rate of proton generation over time at varying substrate concentrations was fit to the Michaelis-Menten equation using MATLAB software to obtain *K*_m_, *V*_max_, and *k*_cat_ values. To evaluate the effects of metal ions on activity, stock solutions of each metal were prepared at 100 mM and adjusted to pH 5.5. Prior to reaction initiation, each metal ion was added to the phenol red reaction mixture to a final concentration of 200 µM. Kinetic analysis of AiiF with varying AHL substitutions was performed under the same conditions, except that C4-HSL variants were added at 5 mM in the presence of 200 µM CoCl₂ only. The initial linear region of proton generation over time was defined as the initial rate and was divided by the enzyme concentration to estimate $k_{obs}$ values.

### pH activity assay

The effect of pH on purified AiiF activity was evaluated using a reaction mixture similar to that described for the metal ion assay, consisting of HEPES/NaCl buffer, 200 µM cobalt, 5 mM C4-HSL, and 100 nM enzyme. The pH of the reaction mixture was adjusted to the desired value using HCl or NaOH prior to initiating the reaction. Reactions were allowed to proceed overnight, after which residual AHL concentrations were quantified using the short-chain AHL biosensor pSB536. Briefly, E. *coli* cells harboring pSB536 were grown to mid-log phase (OD₆₀₀ = 0.3–0.6). Subsequently, 190 µL of biosensor culture was combined with 10 µL of the reaction mixture and incubated in a microplate reader at 37°C for 8 h. Luminescence and OD₆₀₀ measurements were recorded every 20 min. Luminescence values were normalized to OD₆₀₀, and the resulting normalized luminescence data were integrated over time for analysis.

### BioLuminate docking

BioLuminate software (Schrödinger Release 2023-1; Schrödinger, LLC, New York, NY, USA) was used to estimate the relative binding affinities of AHLs to the AiiF structure in the presence of cobalt. The Protein Preparation Wizard was used to assign bond orders, add missing side chains, and optimize water geometry. Docking grids were centered on the metal ions, with inner grid box dimensions set to x = 10 Å, y = 10 Å, and z = 8 Å, and outer grid box dimensions set to x = 30 Å, y = 30 Å, and z = 30 Å. Three-dimensional AHL structures were downloaded from PubChem as SDF files and prepared using LigPrep. Ligand docking was performed using Glide, where metal coordination constraints, ligand flexibility, and enhanced sampling settings were applied (93). Docking output data were analyzed using the Project Table within BioLuminate. Two-dimensional ligand interaction diagrams were generated using the Structural Analysis module in BioLuminate.

### Biofilm formation assay

The biofilm formation assay was performed as previously described (38). Briefly, cultures of *Acidithiobacillus ferrooxidans* and *Acidithiobacillus thiooxidans* were inoculated 1:100 into clear 96-well plates to a final volume of 200 µL containing SM4 or Starkey medium, respectively (94, 95). Both media were adjusted to pH 5 and supplemented with 0.1% (wt/vol) dispersed sulfur (96). Wells were supplemented daily with 10 µM protein or buffer control. Following 2 days of incubation at 30°C, planktonic cells were gently removed, and adherent biofilms were stained with 0.1% crystal violet solution at room temperature for 15 min. Excess stain was removed by submerging the plate in sterile DI water until unbound crystal violet was eliminated. Bound crystal violet was solubilized by adding 100 µL of 95% ethanol to each well and incubating for 15 min. All crystal violet steps were performed under dark conditions. Biofilm biomass was quantified by measuring absorbance at 560 nm using a microplate reader. *Acidithiobacillus ferrooxidans* type strain ATCC 23270 and *Acidithiobacillus thiooxidans* type strain ATCC 19377 were obtained from the American Type Culture Collection (ATCC, Manassas, VA, USA).

### Engineering of *Acidithiobacillus thiooxidans*

The AiiF gene was inserted into a pJRD215 plasmid with superfolder GFP fused to its N-terminus under a constitutive tac promoter using primers listed in Table S4 (57, 97). *A. thiooxidans* cells were engineered as previously described (98). Briefly, starter cultures grown in Starkey medium with 1% colloidal sulfur were harvested once the pH reached 1.5 and centrifuged at 5,000 × *g* for 7 min to pellet cells. Subsequent spins at 3,000 × *g* for 10 s removed residual sulfur fractions before re-pelleting at 17,000 × *g* for 1 min. Cell pellets were washed sequentially with phosphate-buffered saline (PBS) and DI water. All solutions were pre-chilled on ice. Cells were resuspended in DI water to achieve an OD₆₀₀ of 5, and 400 µL aliquots were transferred into pre-chilled 2 mm gap electroporation cuvettes. A total of 60 ng of plasmid DNA was added to each cuvette prior to electroporation. Electroporation was performed at 2.5 kV with a resistance of 400 Ω and a time constant of 5 ms. Cells were immediately recovered in ice-cold Starkey medium (pH 5) supplemented with 0.1% dispersed sulfur with shaking at 30°C. Kanamycin was added to a final concentration of 200 µg/mL 12 h post-electroporation, and transformants were selected by five rounds of subsequent antibiotic passaging. The electroporation was confirmed by colony PCR and gel electrophoresis (Fig. S7) using primers listed in Table S4.

Protein expression (GFP, GFP-AiiF) in *Acidithiobacillus thiooxidans* was confirmed using flow cytometry with a NovoCyte Quanteon cell analyzer (Agilent). Green fluorescent protein fluorescence was measured using the B525 channel with an excitation of 488 and an emission of 525/40 at a detector gain of 564. Samples were acquired with 100 µL stop conditions at a flow rate of 50 µL/min. Data were analyzed using NovoCyte Express software, and single cells were gated based on forward- and side-scatter profiles.

### *A. thiooxidans* growth curves

Engineered AT-GFP and AT-GFP-AiiF cultures were grown to late exponential phase and inoculated 1:10 into Starkey media adjusted to pH 5 with 2 g/L of dispersed sulfur, shaking at 30°C. An abiotic control contained uninoculated medium under the same conditions. Daily or bi-daily pH measurements were taken using a pH probe, with new calibrations performed before each measurement.

### SEM biofilm imaging

Sulfur coupons were submerged in Starkey medium (pH 5) containing a 1:10 inoculum of *Acidithiobacillus thiooxidans* strains harboring either AiiF-GFP or GFP control plasmids, or uninoculated medium in triplicate. Cultures were grown in stationary 96-well plates at 30°C for 3 days. Following incubation, sulfur coupons were removed using sterile forceps and gently washed twice with PBS. Coupons were then immersed in 4% paraformaldehyde in PBS (Bioworld, Dublin, OH, USA) for 2 h at room temperature. The fixative was removed by washing the coupons twice with PBS. Dehydration was performed using a graded ethanol series, with coupons incubated for 10 min each in 30%, 50%, 70%, 80%, 90%, 95%, and 100% ethanol. The 100% ethanol incubation step was repeated twice. After drying, coupons were mounted onto carbon tape and sputter-coated with approximately 4 nm of gold. Samples were imaged using a Zeiss Sigma VP scanning electron microscope operated according to the manufacturer’s instructions at an accelerating voltage (EHT) of 2 kV.

### Statistics

Statistical significance was evaluated using either one-way Brown-Forsythe and Welch ANOVA tests or ordinary one-way ANOVA, assuming Gaussian distributions and unpaired samples. Post hoc Tukey multiple-comparison tests were performed following ordinary one-way ANOVA, where appropriate. Statistical significance was defined as *P* < 0.05. All statistical analyses were conducted using GraphPad Prism.

## SUPPLEMENTAL MATERIAL

Supplemental material aem.00758-26-s0001.pdf Tables S1 to S4; Fig. S1 to S7.

ASM does not own the copyrights to Supplemental Material that may be linked to, or accessed through, an article. The authors have granted ASM a non-exclusive, world-wide license to publish the Supplemental Material files. Please contact the corresponding author directly for reuse.
