# Supplementary material for: Identification and characterization of quorum-quenching enzyme AiiF from Acidithiobacillus ferrooxidans and its application for biofilm disruption
Source: Appl Environ Microbiol. 2026 Jul 17;92(8):e00758-26. doi: 10.1128/aem.00758-26 (PMC13488345; doi:10.1128/aem.00758-26)
Supplement: Supplementary file 1 [file aem.00758-26-s0001.pdf]

Supplemental Materials

for

Identification and characterization of a quorum quenching enzyme AiiF from *Acidithiobacillus ferrooxidans* and its application for biofilm disruption

Mallori A. Herishko, Hannah S. Zurier, and Scott Banta*

Department of Chemical Engineering, Columbia University, New York, NY 10027

* Corresponding author. E-mail: sbanta@columbia.edu

Supplementary Tables:

Table S1. List of all protein names, sequences, and reference numbers used in the sequence alignments and comparisons

Protein Name	Amino Acid Sequence	Reference Number
AiiF	MTQVLKMWPLLTATHHYDKSISTRHRGQGQVIEAPILA<br>YLIETKHGRILYDVGCDYGKIADQQQRDRYYD<br>SKIFGLEAPNMQEGQRLHHHLERLGLTSADVDLVFLGH<br>LHFDHAGGLCEVCGAEVHVQSDELEAANSGLD<br>PAYFPDDFSGDYRWKVMSGEYDLVPGVRALSSPGHTA<br>GHMSLWVELPHGAPVILCGDAADLEENLRDEIA<br>PGICWQDSEEQALASIRKLKHLAEQEKAQLWPNHDIAF<br>WQTLRQFPDFHD	WP_163059719.1
AiiA	MTVKKLYFVPAGRCMLDHSSVNSTLTPGELLDLPVWC<br>YLLETEEGPILVDTGMPESAVNN<br>EGLFNGTFVEGQVLPKMTEEDRIVNILKRVGYEPEDLLY<br>IISSHLHFDHAGGNGAFINTP<br>IIVQRAEYEAAQHSEEYLKECILPNLNYKIIEGDYEVVPG<br>VQLLHTPGHTPGHQSLLIET<br>EKSGPVLLTIDASYTKENFENEVPFAGFDSELALSSIKRL<br>KEVVMKEKPIVFFGHDIEQE<br>RGCKVFPEYI	Q9L8R8
AttM	MTDIRLYMLQSGTLKCKVHNIKMNQGNGADYEIPVPFF<br>LITHPAGHTVIDGGNAIEVATD<br>PRGHWGGICDVYWPVLDKDQGCVDQIKALGFDPADVK<br>YVVQSHLHLDHTGAIGRFPNATH<br>IVQRSEYEYAFTPDWFAGGGYIRKDFDKPGLKWQFLNG<br>AQDDYYDVYGDGTLTTIFTPGH<br>APGHQSFLVRLPNSKPLLLTIDAAYTLDHWEEKALPGFL<br>ASTVDTVRSVQKLRTYAEKHD<br>ATVVTGHDPDAWANFKKAPEFYA	Q7D3U0
AiiB	MGNKLFVLDLGEIRVDENFIIANSTFVTPQKPTVSSRLID<br>IPVSAYLIQCTDATVLYDTG<br>CHPECMGTNGRWPAQSQLNAPYIGASECNLPERLRQLG<br>LSPDDISTVVLSHLHNDHAGCV<br>EYFGKSRLIAHEDEFATAVRYFATGDHSSPYIVKDIEAW<br>LATPRNWDLVGRDERERELAP<br>GVNLLNFGTGHASGMLGLAVRLEKQPGFLLVSDACYT<br>ATNYGPPARRAGVLHDTIGYDRT<br>VSHIRQYAESRSLTVLFGHDREQFASLIKSTDGFYE	A9CKY2
MomL	MNMKKLLVLLLVVGAFSCKQAKKEAAESETAVTEAAK<br>PEIKLYAFSGGTVNANMLELFSQDTTYTGQSKE<br>FADAFYVIVHPKGTLMWDAGLPESLVGLPEPFTSPDGA<br>FTVSRKDSVANQLASIDMTVDDIDFIALSHTH	AIY30473.1

	FDHIGHANVFAGSTWLVQEKEYDFVTSEDNQKSNPDIY<br>NSIKELTKVKKINGDYDVFGDGSVVMKFMPGH<br>TPGHQVLYLDMVEHGPLMLSGDMYHFYENREFRRVPIF<br>NYDVALTKKSMGEFEAFAEEKGAKVYLQHSKE<br>DFEKLPQAPNYLQ	
AaL	MTNIAKAQPKLYVMDNGRMRMDKNWMIAMHNPATIA<br>NPNAPTEFIEFPIYTVLIDHPEGKILFDTSCNPD<br>SMGAQGRWGEATQSMFPWTASEECYLHNRLEQLKVRP<br>EDIKFVIASHLHLDHAGCLEMFTNATIIVHEDE<br>FSGALQTYARNQTEGAYIWGDIDAWIKNNLNWRTIKR<br>DEDNIVLAEGIKILNFGSGHAWGMLGLHVQLPE<br>KGGIILASDAVYSAESYGPPIKPPGIIYDSLGFVRSVEKIK<br>RIAKETNSEVWFGHDSEQFKRFRKSTEGY<br>YE	WP_021296945.1
AidP	MTGIIKPTPKLYVMDNGTLSMDKNFMISMHNPASIENP<br>NQPSEMATFPVYTVLIDHPEGKILFDTACNPN<br>SMGAEGRWGKVTQSLFPIDMPEECYLHNRLEELGVRPE<br>DIKYVVASHLHLDHAGCLELFTNATIIVHEDE<br>LNGALQTYARNTKEGAYIWGDIDAWIKNNLQWKTVKR<br>TEDGLQLAEGIKLLNFGSGHAWGMLGLQIQLPE<br>TGGIILASDAIYTAESFGPPVKPPGILYDSVGYTSAVEKIR<br>RLSQETNSDVWFGHDANQFKTFRKSTEGY<br>YE	WP_049694637.1
AiiK	MCQNKKLYVLDTGTMKMDKNYMIAMHNPATIDAPTK<br>PAEFVEFPVYAVLIDHPEGKILFDTGCNPDSMNE<br>DGPWPEGIRRAFPTFATEECYLINRLEQLKVRPEDIKYV<br>VASHLHLDHAGCLELFTNAEIIVHDAELASV<br>MKSFAMTRDMGAYIWADVMSWVQKQLRWRTVKVW<br>QKELQLVEGIKIINFGPGHAYGMLGLHVELPGYGNV<br>LLASDAVYTKESYGPPIKPPGILYDSVGYNQTVERIHQF<br>AHEHKSEVWFGHDAEQFKSFRKSTEGYYE	WP_029500404.1
GcL	MANVIKARPKLYVMDNGRMRMDKNWMIAMHNPATIH<br>NPNAQTEFVEFPIYTVLIDHPEGKILFDTSCNPN<br>SMGPQGRWAESTQQMFPWTATEECYLHNRLEQLKVRP<br>EDIRYVVASHLHLDHAGCLEMFTNATIIVHEDE<br>FNGALQCYARNQKEGAYIWADIDAWIKNNLQWRTVK<br>RHEDNILLAEGVKVLNFGSGHAWGMLGLHVELPE<br>TGGIILASDAIYTAESYGPPIKPPGIIYDSLGYMNTVERIR<br>RIAQETKSQVWFGHDAEQFKKFRKSTEGY<br>YE	WP_017434252
AidH	MEESRNIIRITNRLTIHEDDLEESFIRSSGPGGQNVNKVST<br>AVQLRFHASQSGLPEDVLSRLFKLAGQKG<br>TKDGDILIEANRFRTQERNREDARERMIALIAKAAEPPPP<br>PRRKTKPTKGSIERRLKAKSGRSDIKKGRG<br>KVSHD	ACZ73822.1

SsoPOX	MRIPLVGKDSIESKDIGFTLIHEHLRVFSEAVRQQWPHL YNEDEEFRNAVNEVKRAMQFGVKTIVDPTVMGLGRDI RFMEKVVKATGINLVAGTGIYIYIDLPFYFLNRSIDEIAD LFIHDIKEGIQGTLNKAGFVKIAADEPGITKDVEKVIRAA AIANKETKVPIITHSNAHNNTGLEQQRILTEEGVDPGKIL IGHLGDTDNIDYIKKIADKGSFIGLDRYGLDLFLPVDKR NETTLRLIKDGYSDKIMISHDYCCTIDWGTAKPEYKPKL APRWSITLIFEDTIPFLKRNGVNEEVIATIFKENPKKFFS	pdb_00003uf9
PON2	MGRLVAVGLLGIALALLGERLLALRNRLKASREVESVD LPHCHLIKGIEAGSEDIDILPN GLAFFSVGLKFPGLHSFAPDKPGGILMMDLKEEKPRAR ELRISRGFDLASFNPHGISTFI DNDDTVYLFVVNHPEFKNTVEIFKFEEAENSLLHLKTV KHELLPSVNDITAVGPAHFYAT NDHYFSDPFLKYLETYLNLHWANVVYYSPNEVKVVAE GFDSANGINISPDDKYIYVADIL AHEIHVLEKHTNMNLTQLKVLELDTLVDNLSIDPSSGDI WVGCHPNGQKLFVYDPNNPPS SEVLRIQNILSEKPTVTTVYANNGSVLQGSSVASVYDGK LLIGTLYHRALYCEL	Q15165

Table S2. Comparison list of kinetic parameters for MBLs with C6-HSL

Protein Name	K_m (mM)	k_{cat} (s^{-1})	k_{cat}/K_m $(M^{-1}s^{-1})$	Reference
AiiF	3.5 ± 2.2 *	0.15 ± 0.04 *	42 ± 29 *	This work
AiiA	3.8 **	36 **	9.3×10^3 **	(1)
AiiB	1.6 ± 0.2	25 ± 0.9	$1.6 \pm 0.3 \times 10^4$	(2)
AaL	0.083 ± 0.1	14 ± 0.4	$1.7 \pm 0.2 \times 10^5$	(3)
MomL	0.79 ± 0.08	230 ± 8	2.9×10^5 **	(4)
AiiK	0.20 ± 0.03	0.65 ± 0.07	3.2×10^3 **	(5)
GcL	0.0080 ± 0.002	9.0 ± 0.5	$1.1 \pm 0.3 \times 10^6$	(6)

*Data for AiiF collected using C4-HSL, not C6-HSL

**Indicates that error was not reported

Table S3. Docking results of AHL into AiiF using BioLuminate are displayed as GlideScore and E Model for each condition. GlideScore represents the approximate ligand binding energy. More negative values indicate stronger binding affinity. E Model scores represent the energy favorability of each AHL to AiiF docking position.

HSL	GlideScore	E Model
C4	-4.07	-39.0
C6	-4.20	-38.0
C8	-1.49	-35.7
C10	-1.80	-40.4
C12	-0.569	-31.5
C14	-2.82	-43.2
3-oxo-C4	-4.13	-39.8
3-oxo-C6	-3.81	-37.3
3-oxo -C8	-3.33	-38.0
3-oxo-C10	-0.818	-29.9
3-oxo-C12	-1.59	-39.0
3-oxo-C14	-2.81	-39.0
3-Hydroxyl-C4	-4.22	-43.2
3-Hydroxyl-C6	-4.76	-50.5
3-Hydroxyl-C8	-2.32	-41.1
3-Hydroxyl-C10	-1.80	-40.4
3-Hydroxyl-C12	-2.59	-46.9
3-Hydroxyl-C14	-3.55	-48.4

Table S4. Plasmid, primers, and proteins for genetic manipulation in the study.

Segment Type	Sequence
Primers	
pMal Vector Forward 5’	TAAAAGCTTGGCACTGGC
pMal Vector Reverse 5’	CTTGTCATCGTCATCCCC
AiiF Insert into pMal Forward 5’	TCGGGGATGACGATGACAAGATGACTCAAGTCCTGAAAATG
AiiF Insert into pMal Reverse 5’	CGGCCAGTGCCAAGCTTTTACTTCTCAAACTGAGGATG
pJRD215 Vector Forward 5’	TACCCCTACGATGTGCCC
pJRD215 Vector Reverse 5’	GTACCATACCTGCTTGAGATATTC
AiiF Insert into pJRD215Forward 5’	GTATGGACGAACTCTATAAGATGACTCAAGTCCTGAAAATG
AiiF Insert into pJRD215 Reverse 5’	GTAGGAGTCAAACTCTTCATGGGGATGCTACACGGGCT
Colony PCR GFP Forward 5’	ATGAGCAAAGGCGAAGAA
Colony PCR GFP Reverse 5’	CTTATAGAGTTCGTCCATACC
Colony PCR AiiF Forward 5’	ATGACTCAAGTCCTGAAAATG

Colony PCR AiiF Reverse 5’	ATCGTGAAAGTCTGGAAAC
Plasmids	
pMal Vector	(7)
pJRD215 Vector	(8)
Proteins	
Superfolder GFP	MSKGEELFTGVVPILVELDGDVNGHK<br>FSVRGEGEGDATNGKLTLKFICTTGK<br>LPVPWPTLVTTLTYGVQCFSRYPDHM<br>KRHDFFKSAMPEGYVQERTISFKDDG<br>TYKTRAEVKFEGDTLVNRIELKGIDF<br>KEDGNILGHKLEYNFNSHNVYITADK<br>QKNGIKANFKIRHNVEDGSVQLADH<br>YQQNTPIGDGPVLLPDNHYLSTQSVL<br>SKDPNEKRDHMVLLEFVTAAGITHG<br>MDELYK
Superfolder GFP-AiiF	MSKGEELFTGVVPILVELDGDVNGHK<br>FSVRGEGEGDATNGKLTLKFICTTGK<br>LPVPWPTLVTTLTYGVQCFSRYPDHM<br>KRHDFFKSAMPEGYVQERTISFKDDG<br>TYKTRAEVKFEGDTLVNRIELKGIDF<br>KEDGNILGHKLEYNFNSHNVYITADK<br>QKNGIKANFKIRHNVEDGSVQLADH<br>YQQNTPIGDGPVLLPDNHYLSTQSVL<br>SKDPNEKRDHMVLLEFVTAAGITHG<br>MDELYKMTQVLKMWPLLTATHHYD<br>KSISTRHRGQGQVIEAPILAYLIETKH<br>GRILYDVGCDYGKIADQQQRDRYYD<br>SKIFGLEAPNMQEGQRLHHHLERLGL<br>TSADVDLVFLGHLHFDHAGGLCEVC<br>GAEVHVQSDELEAANSGLDPAYFPD

	DFSGDYRWKVMSGEYDLVPGVRALS<br>SPGHTAGHMSLWVELPHGAPVILCGD<br>AADLEENLRDEIAPGICWQDSEEQAL<br>ASIRKLKHLAEQEKAQLWPNHDIAFW<br>QTLRQFPDFHDWSHPQFEKYPYDVPD<br>YA

Supplementary Figures:

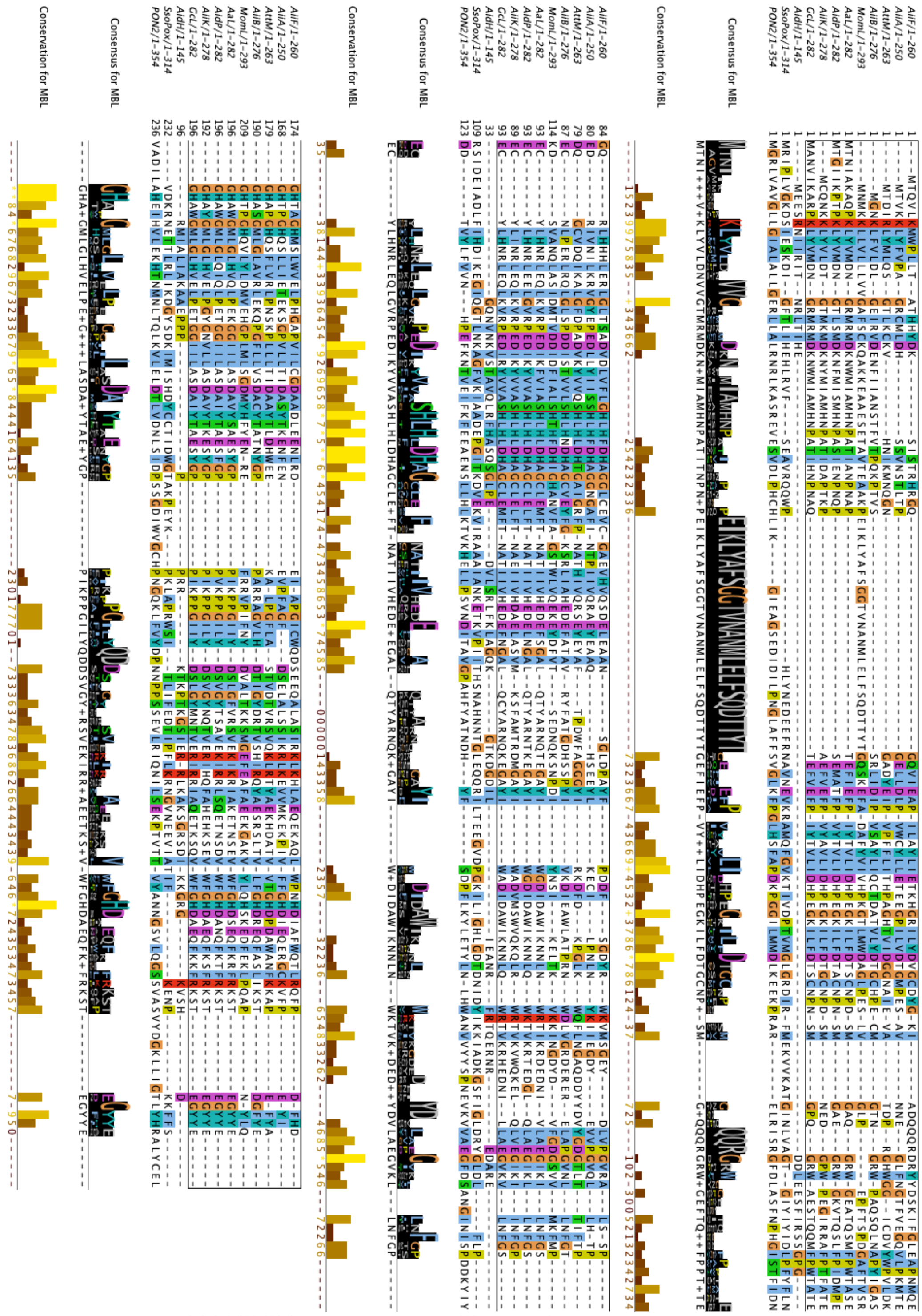

Figure S1. Jalview sequence alignment of MBLs. Amino acids are color coded using Clustal, where: blue indicates hydrophobic, red indicates positive charge, magenta indicates negative charge, green indicates polar, pink indicates cysteines, orange indicates glycines, yellow indicates prolines, cyan indicates aromatic, and white indicates unconserved.

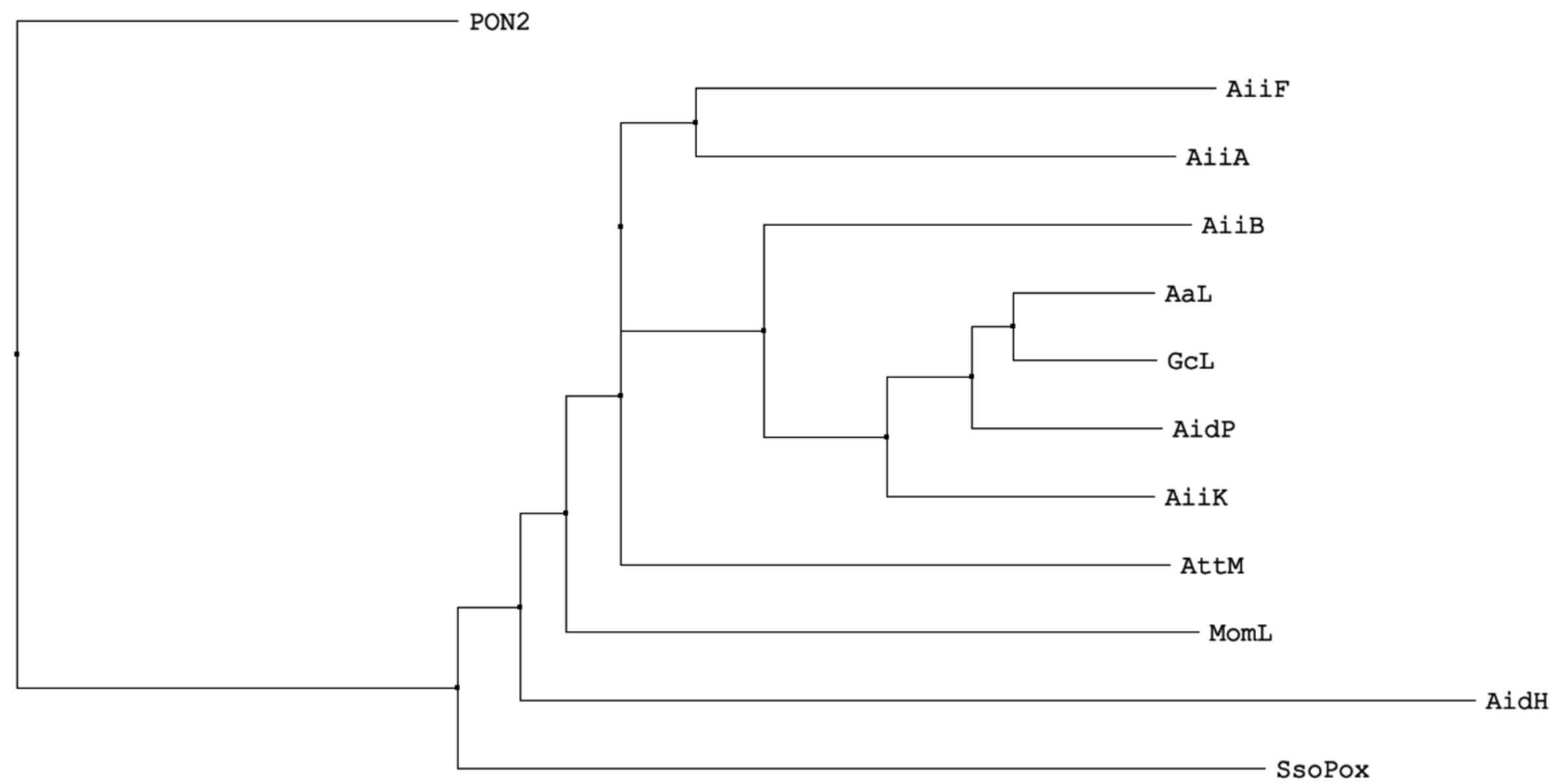

Figure S2. Phylogenetic tree was constructed using Blosum62 in Jalview to highlight sequence similarity and placement of representative MBL relative to AiiF, not to show evolutionary uniqueness amongst acidophiles.

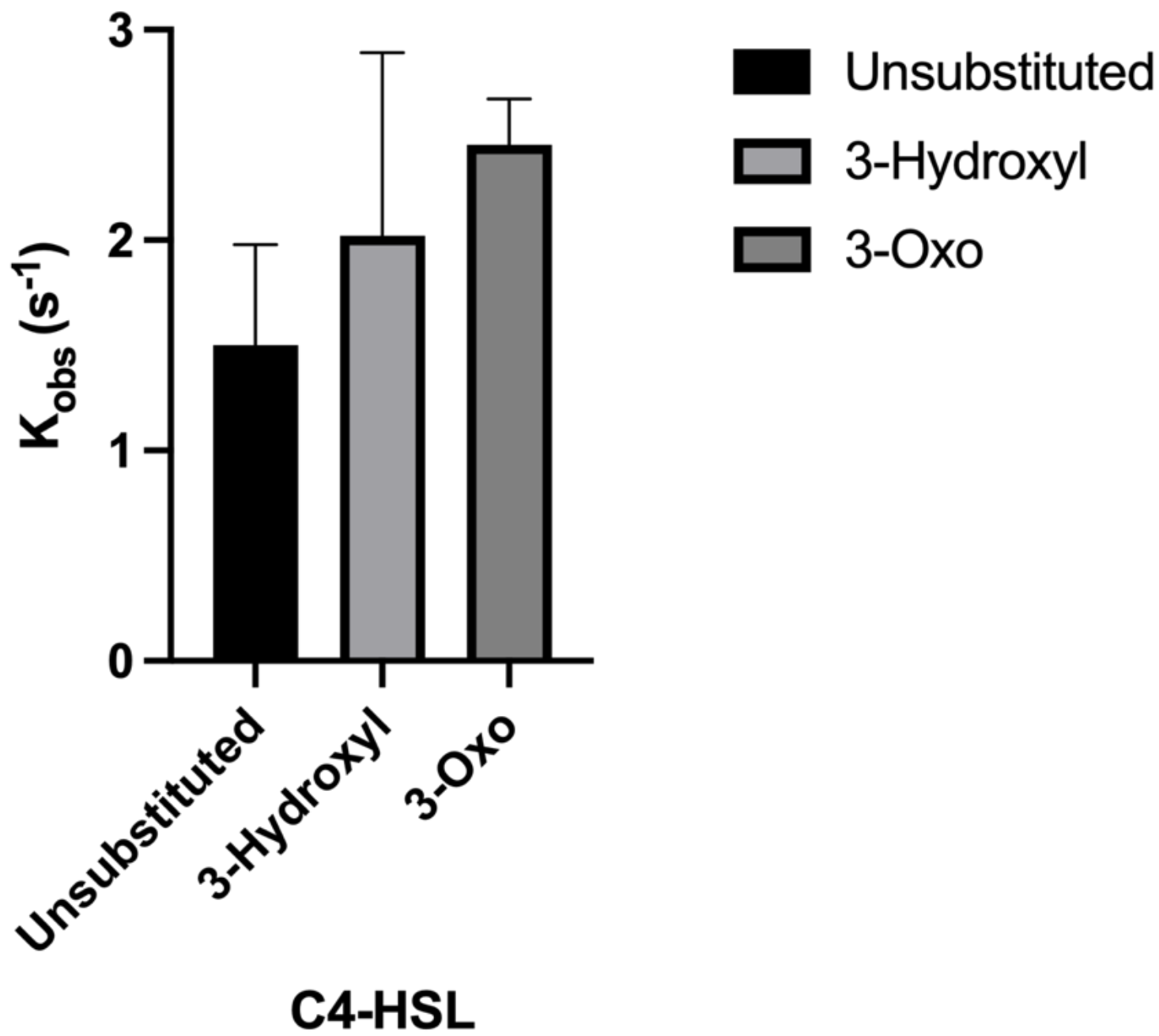

Figure S3. k_{obs}values for reactions between AiiF and various substitutions of 5 mM C4-HSL. Bars represent averages of k_{obs} values obtained via phenol red kinetic assays. All experimements were performd in triplicates, and error bars represent standard deviation with Ordinary one-way ANOVA with Tukey's multiple comparisons test indicate that $p > 0.05$ for all cases.

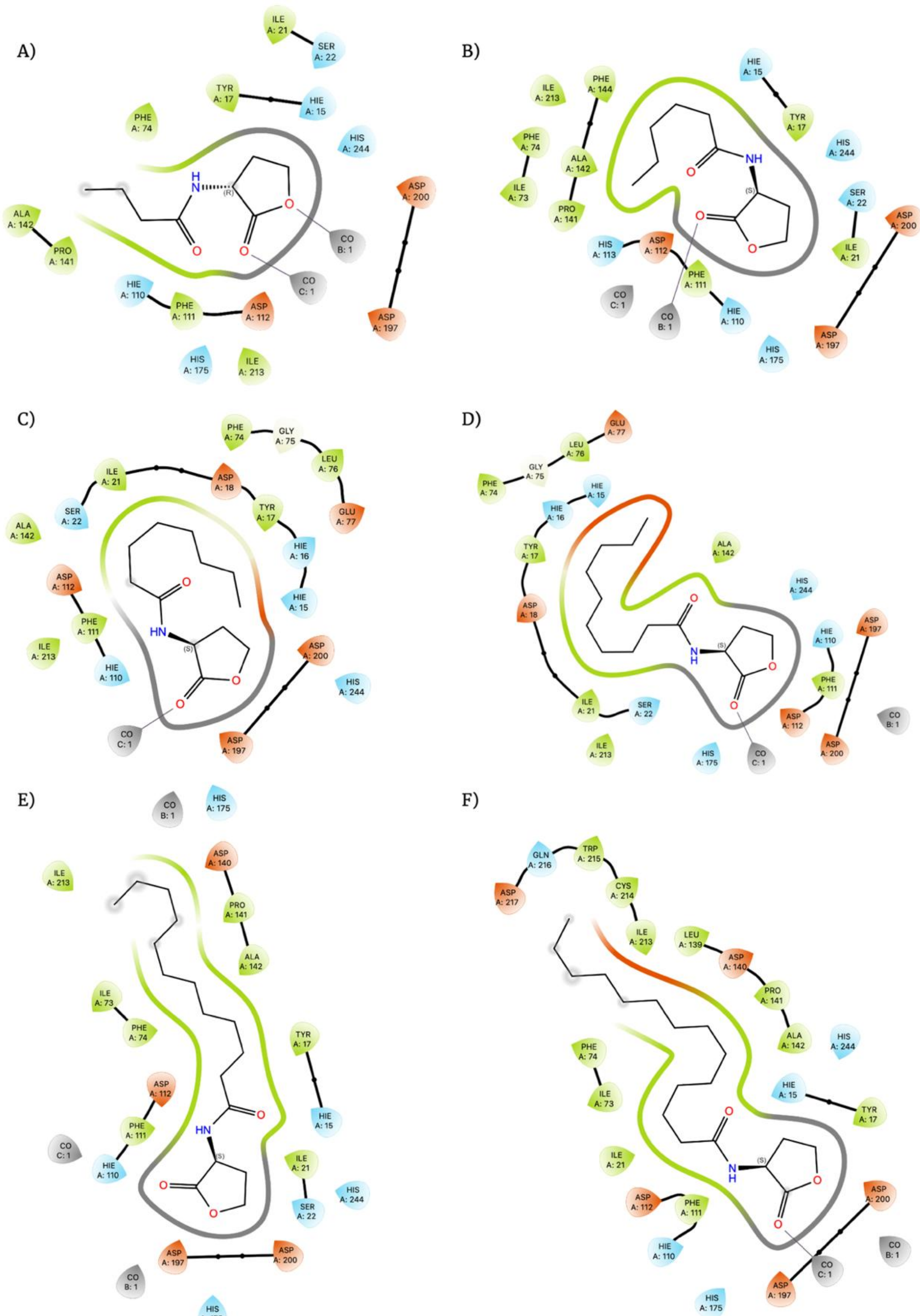

Figure S4. Ligand Interaction Diagram of AiiF to A) C4-HSL B) C6-HSL C) C8-HSL D) C10-HSL E) C12-HSL and F) C14-HSL generated using Schrödinger's BioLuminate Glide ligand-interaction diagrams. Red indicates negatively charged residues, blue indicates polar residues, green lines indicate hydrophobic residues, grey indicates metal or solvent exposure. Straight purple lines indicate metal coordination.

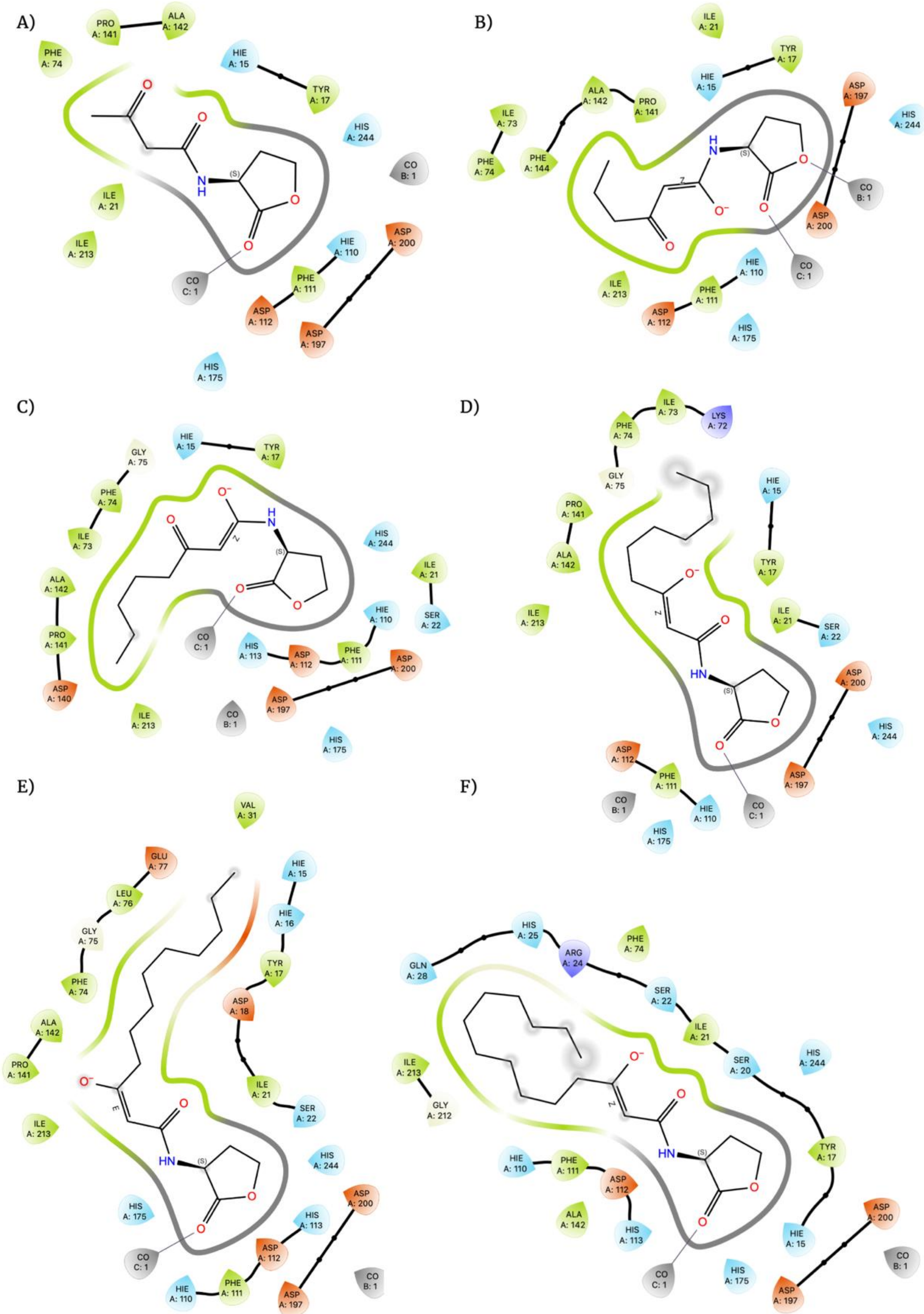

Figure S5. BioLuminate-generated ligand-interaction diagrams for 3-Oxo-HSLs: A) C4, B) C6, C) C8, D) C10, E) C12, F) C14. The color scheme representation can be found in Figure S4

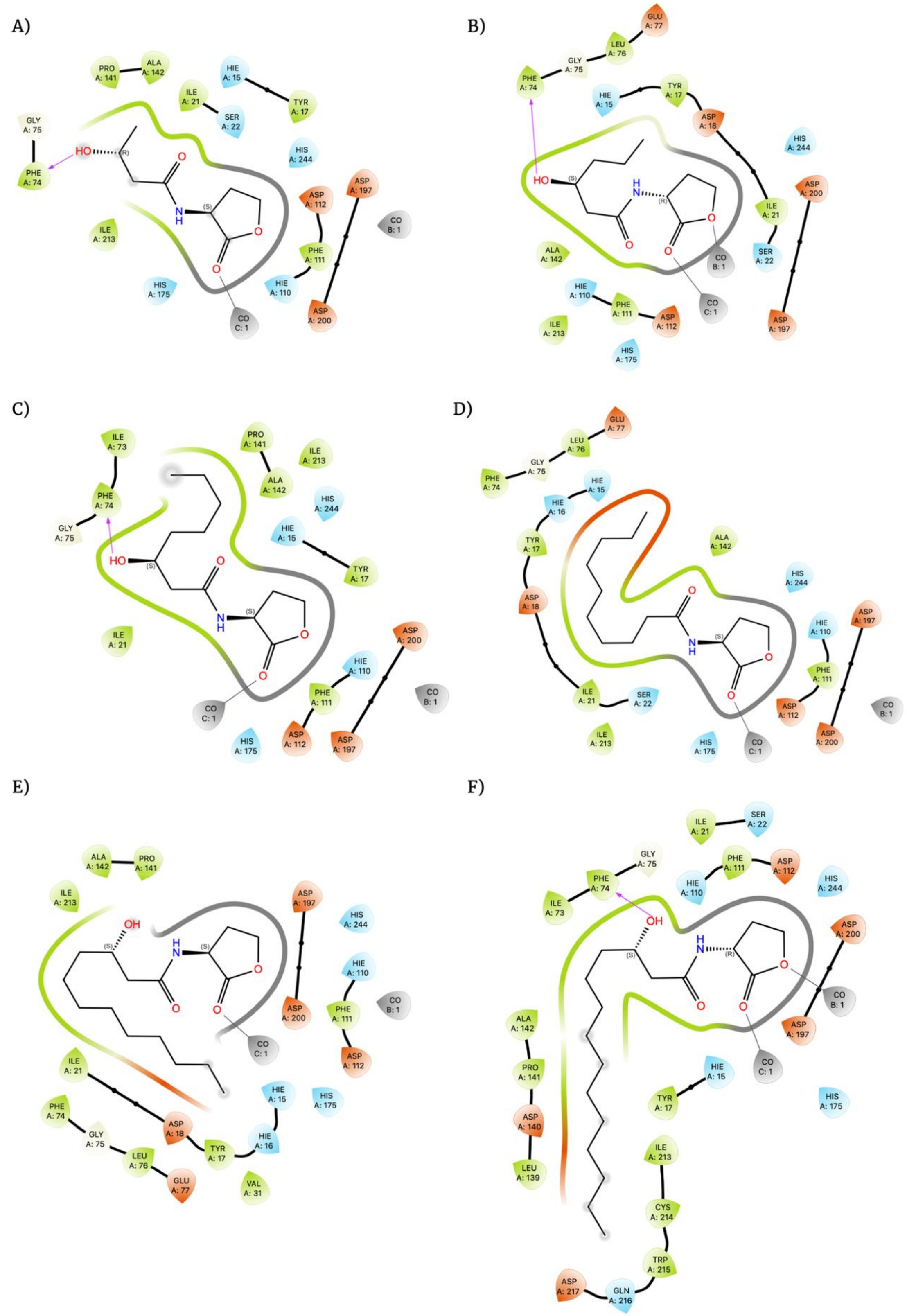

Figure S6. BioLuminate-generated ligand-interaction diagrams for 3-Hydroxyl-HSLs: A) C4, B) C6, C) C8, D) C10, E) C12, F) C14. The color scheme representation can be found in Figure S4.

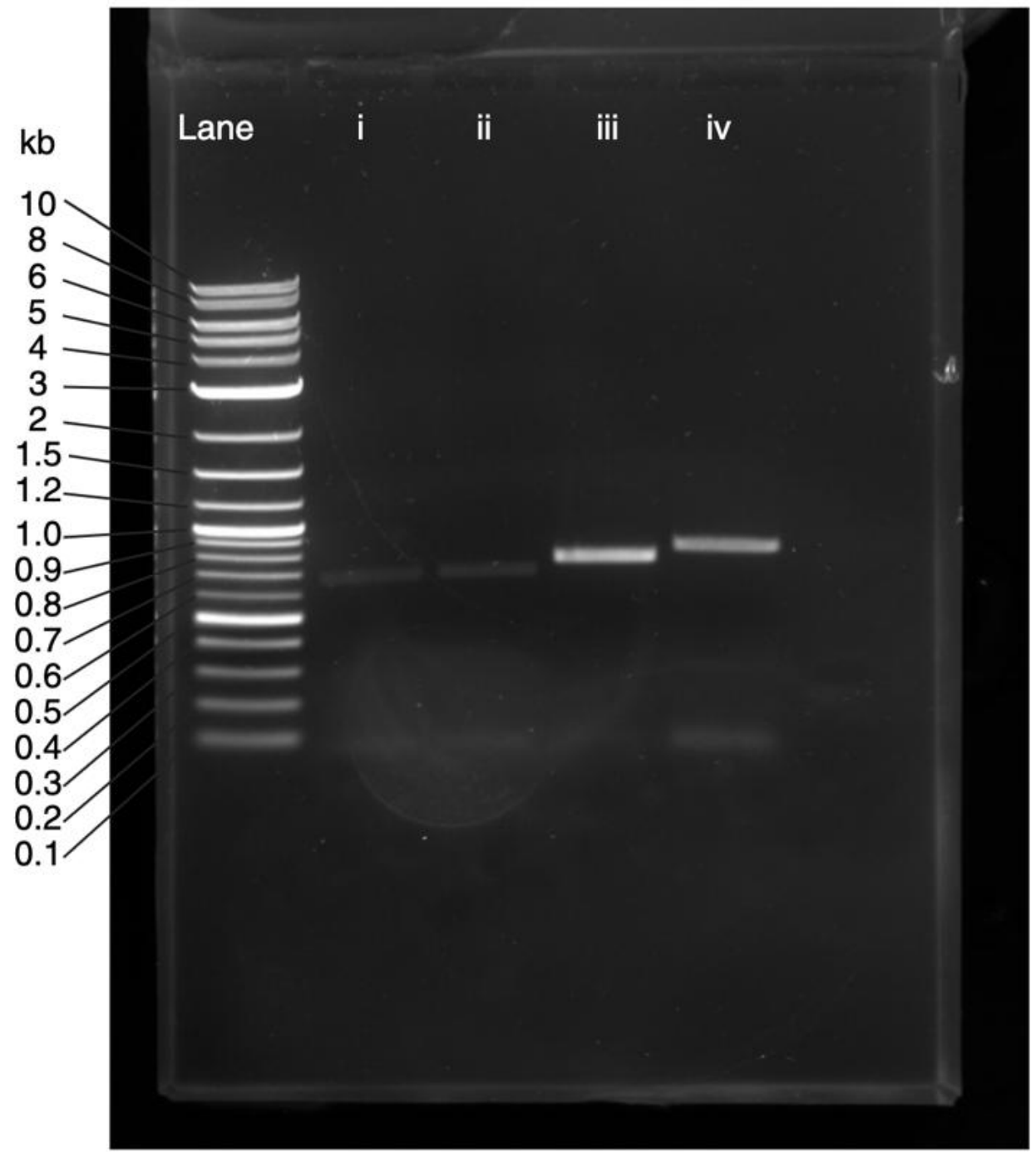

Figure S7. Agarose electrophoresis of DNA fragments generated from the colony PCR of engineered A. *thiooxidans* strains using primers targeting i) GFP of PYI14-GFP (714 bp), ii) GFP of PYI14-GFP-AiiF (714 bp), iii) enzyme not included in this study, and iv) AiiF of PYI14-GFP-AiiF (780 bp) using primers listed in Table S4.

References:

1. Wang L-H, Weng L-X, Dong Y-H, Zhang L-H. 2004. Specificity and enzyme kinetics of the quorum-quenching N-acyl homoserine lactone lactonase (AHL-lactonase) *. Journal of Biological Chemistry 279:13645–13651.

2. Liu D, Thomas PW, Momb J, Hoang QQ, Petsko GA, Ringe D, Fast W. 2007. Structure and specificity of a quorum-quenching lactonase (AiiB) from *Agrobacterium tumefaciens*. Biochemistry 46:11789–11799.

3. Bergonzi C, Schwab M, Naik T, Daudé D, Chabrière E, Elias M. 2018. Structural and biochemical characterization of AaL, a quorum quenching lactonase with unusual kinetic properties. Sci Rep 8:11262.

4. Tang K, Su Y, Brackman G, Cui F, Zhang Y, Shi X, Coenye T, Zhang X-H. 2015. MomL, a novel marine-derived N-acyl homoserine lactonase from *Muricauda olearia*. Applied and Environmental Microbiology 81:774–782.

5. Dong W, Zhu J, Guo X, Kong D, Zhang Q, Zhou Y, Liu X, Zhao S, Ruan Z. 2018. Characterization of AiiK, an AHL lactonase, from *Kurthia huakui* LAM0618T and its application in quorum quenching on *Pseudomonas aeruginosa* PAO1. Sci Rep 8:6013.

6. Bergonzi C, Schwab M, Elias M. 2016. The quorum-quenching lactonase from *Geobacillus caldoxylosilyticus*: purification, characterization, crystallization and crystallographic analysis. Acta Crystallogr F Struct Biol Commun 72:681–686.

7. Khoury F, Su Z, Banta S. 2024. Rare earth element binding and recovery by a beta roll-forming RTX domain. Inorg Chem 63:13223–13230.

8. Davison J, Heusterspreute M, Chevalier N, Ha-Thi V, Brunei F. 1987. Vectors with restriction site banks V. pJRD215, a wide-host-range cosmid vector with multiple cloning sites. Gene 51:275–280.
